# The Perils of Navigating Activity-Dependent Alternative Splicing of Neurexins

**DOI:** 10.3389/fnmol.2021.659681

**Published:** 2021-03-09

**Authors:** Kif Liakath-Ali, Thomas C. Südhof

**Affiliations:** ^1^Howard Hughes Medical Institute, Stanford University, Stanford, CA, United States; ^2^Department of Molecular and Cellular Physiology, Stanford University, Stanford, CA, United States

**Keywords:** neurexins, alternative splicing, cFos, cell dealth, depolarization, kainate, cerebellum, hippocampus

## Abstract

Neurexins are presynaptic cell-adhesion molecules essential for synaptic function that are expressed in thousands of alternatively spliced isoforms. Recent studies suggested that alternative splicing at splice site 4 (SS4) of Nrxn1 is tightly regulated by an activity-dependent mechanism. Given that Nrxn1 alternative splicing at SS4 controls NMDA-receptor-mediated synaptic responses, activity-dependent SS4 alternative splicing would suggest a new synaptic plasticity mechanism. However, conflicting results confound the assessment of neurexin alternative splicing, prompting us to re-evaluate this issue. We find that in cortical cultures, membrane depolarization by elevated extracellular K^+^-concentrations produced an apparent shift in Nrxn1-SS4 alternative splicing by inducing neuronal but not astroglial cell death, resulting in persistent astroglial Nrxn1-SS4+ expression and decreased neuronal Nrxn1-SS4– expression. *in vivo*, systemic kainate-induced activation of neurons in the hippocampus produced no changes in Nrxn1-SS4 alternative splicing. Moreover, focal kainate injections into the mouse cerebellum induced small changes in Nrxn1-SS4 alternative splicing that, however, were associated with large decreases in Nrxn1 expression and widespread DNA damage. Our results suggest that although Nrxn1-SS4 alternative splicing may represent a mechanism of activity-dependent synaptic plasticity, common procedures for testing this hypothesis are prone to artifacts, and more sophisticated approaches will be necessary to test this important question.

## Introduction

Neurexins are presynaptic cell-adhesion molecules that play crucial role in defining synapse properties through differential interactions with multifarious extra- and intra-cellular ligands. Genetic perturbation of neurexins and their ligands are implicated in multiple neuropsychiatric disorders (Sudhof, 2017; Kasem et al., 2018; Gomez et al., 2021). Neurexin genes (*Nrxn1, Nrxn2*, and *Nrxn3* in mice) use alternative promotors to transcribe distinct isoforms (α, β, γ), whose mRNAs are subject to extensive alternative splicing in patterns that are specific to neuronal cell types (Treutlein et al., 2014; Fuccillo et al., 2015; Furlanis et al., 2019; Lukacsovich et al., 2019). Six canonical sites of alternative splicing (SS1–SS6) are known, of which SS1, SS2, SS3, and SS6 are specific to α-neurexins, whereas SS4 and SS5 exist in both α- and β-neurexins (Ullrich et al., 1995).

Among the sites of neurexin alternative splicing, SS4 has been most intensely studied. The two alternatively spliced SS4 variants either contain (SS4+) or lack a 90 bp sequences that is encoded by the alternatively spliced SS4 exon (Tabuchi and Sudhof, 2002). SS4– neurexins bind to LRRTMs (Sugita et al., 2001; Ko et al., 2009; Siddiqui et al., 2010; Boucard et al., 2012), whereas SS4+ neurexins bind to cerebellins (Uemura et al., 2010; Matsuda and Yuzaki, 2011). Both SS4+ and SS4– neurexins bind to neuroligins, albeit with differential affinities (Boucard et al., 2005; Chih et al., 2006; Comoletti et al., 2006). In hippocampal synapses, presynaptic Nrxn1 and Nrxn3 SS4 splice variants control, respectively, the postsynaptic NMDA- and AMPA-receptor content by a trans-synaptic mechanism (Aoto et al., 2013; Dai et al., 2019). Thus, at least for some synapses, neurexin SS4 alternative splicing is of central importance in controlling synapse properties.

Several studies reported that alternative splicing of neurexins is activity-dependent (Gorecki et al., 1999; Rozic-Kotliroff and Zisapel, 2007; Iijima et al., 2011; Rozic et al., 2011; Ding et al., 2017). Gorecki et al., were the first to investigate the regulation of neurexin alternative splicing using kainic acid (KA) and pentylenetetrazole (PTZ) stimulation, but concluded that alternative splicing of Nrxn2 at SS1–SS4 was not activity-dependent (Gorecki et al., 1999). Subsequently, Rozic-Kotliroff and Zisapel (2007) and Rozic et al. (2011) showed that Nrxn2 and Nrxn3 alternative splicing at SS3 is modulated by neuronal depolarization induced by high KCl (50 mM). Rozic et al. (2011) also showed that altnernative splicing of all three neurexins at SS4 is modulated in the rat hippocampus by fear conditioning (Rozic-Kotliroff and Zisapel, 2007; Rozic et al., 2011). Furthermore, Iijima et al. (2011) used mild KCl depolarization (25 mM), KA (50 μM), or electrical field stimulation in cultured mouse cerebellar granular cells to demonstrate an increase in Nrxn1 and Nrxn2 SS4– mRNA as a function of activity, whereas Nrxn3 mRNA was unaffected. Iijima et al. (2011) also found a significant increase in Nrxn1-SS4– mRNA abundance when a cerebral hemisphere was exposed to KA through focal injection. In a more extensive recent study, finally, Ding et al. (2017) showed that neuronal activity induces histone modifications that in turn regulate Nrxn1 SS4 alternative splicing. In this astonishing study using KCl-induced depolarization, optogenetic stimulation, and fear conditioning, Ding et al. (2017) uncovered robust changes in Nrxn1 SS4 alternative splicing in mice by various forms of neuronal activity, and confirmed these changes using RNA-seq experiments.

Altogether, especially the more recent papers paint a compelling picture of activity-dependent regulation of neurexin alternative splicing at SS4, the only site of neurexin alternative splicing that has been shown to be physiologically relevant. These results are of great potential significance because they suggest a novel mechanism of synaptic plasticity. However, the various papers report quite variable results and use diverse treatment conditions. Moreover, KCl and KA treatment are known to be neurotoxic (Sun et al., 1992; Cheng and Sun, 1994; Pollard et al., 1994; Kasof et al., 1995; Simonian et al., 1996; Cheung et al., 1998; Takahashi et al., 1999; Gluck et al., 2000; Milatovic et al., 2002; Le Duigou et al., 2005; Wang et al., 2005; Rienecker et al., 2020), thus raising the questions of validity. To address these issues, we here re-evaluated the activity-mediated regulation of Nrxn1 SS4 alternative splicing using simple *in vitro* and *in vivo* paradigms that replicate those employed by Iijima et al. (2011) and Ding et al. (2017), and we extended these experiments to Nrxn2 and Nrxn3 SS4 splice forms. Our data suggest that most, if not all, apparent activity-dependent alternative splicing of Nrxn1 at SS4 induced by high KCl or by KA is produced by the loss of Nrxn1 from neurons that are damaged by the unphysiological KCl or KA stimulation. Since astrocytic Nrxn1-SS4+ remains when neuronal Nrxn1-SS4– is lost, the neuronal cell death creates an apparent shift in Nrxn1 SS4 alternative splicing, thus eliciting the impression of regulated alternative splicing. Although our data do not exclude the possibility of activity-dependent Nrxn1 alternative splicing under other conditions, they suggest that previous reports of such events may have been confounded by the neurotoxic effects of the stimulation paradigms that were employed.

## Methods

### Mice

All animal experiments performed in this study were conducted in accordance with protocols approved by the Administrative Panel on Laboratory Animal Care at Stanford University. Wild type mice used in this study were outbred CD-1 or C57BL/6J [Crl: CD1(ICR) and C57BL/6J mice obtained from Charles River]. Baf53b-Cre [also known as Actl6b-Cre; Tg(Actl6b-Cre)4092Jiwu/J; Stock No: 027826], and RiboTag [B6J.129(Cg)-*Rpl22*^*tm*1.1*Psam*^/SjJ, Stock No: 029977] mice were obtained from the Jackson Laboratory. Female homozygous Baf53b-Cre mice were used to breed with RiboTag males in order to avoid male germline recombination of RiboTag allele. Genotyping was performed using primers prescribed by the animal supplier.

### Primary Cortical Neuronal Culture and Depolarization Experiments

Primary cortical culture from mouse embryos and KCl-mediated depolarization experiment were performed as described in Ding et al. (2017). Briefly, E16.5 embryos were harvested from WT C57B/L6J or CD-1 mice. Cortices were digested using 0.125% trypsin (Thermo Fisher, 15090046) with 0.05% DNase 1 (DN25, Sigma). Dissociated cells were plated on coverslips pre-coated with poly-d-lysine (Sigma, P7280) and laminin (Sigma, 11243217001) in the presence of neuronal platting medium [consist of neurobasal medium (Thermo Fisher), 10% fetal bovine serum (FBS), 1% Glutamax (Thermo Fisher, 35050-061), and penicillin/streptomycin (Thermo Fisher, 15070-63)]. After 3 h of incubation, platting medium was completely removed and cells were supplied with neuronal growth medium [consist of neurobasal medium supplemented with B27 (Thermo Fisher, 17504044), 1% Glutamax, and penicillin/streptomycin]. Media was changed every 3 days by removing 50% old media and replaced with fresh warm new media. Cells were maintained for up to 14 days. For Ara-C treatments, 2 μM cytosine arabinoside (Ara-C) was added to the cells when media was changed.

For KCl-mediated depolarization, the final depolarization buffer consisted 31% depolarization buffer (170 mM KCl, 2 mM CaCl_2_, 1 mM MgCl_2_, 10 mM HEPES), 50% old neuronal conditioned medium and 19% fresh neuronal medium. For 10 to 50 mM KCl treatment experiments, KCl concentration in the depolarization buffer was adjusted accordingly. DIV11 neuronal cultures were treated with the final depolarization solution or NaCl buffer (170 mM NaCl, 2 mM CaCl_2_, 1 mM MgCl_2_, 10 mM HEPES) for 10 min and washed and replaced with old neuronal medium. The culture plate was kept in an incubator and samples were collected at various time points. For chronic KCl exposure, depolarization buffer was left continuously until the samples were collected at different time points.

### Pure Cortical Glial Culture

Embryos at E16.5 day from WT CD-1 mice used for culturing pure cortical glial cells. Cortical cells were subjected to harsh trituation to ensure low number of viable neurons (McCarthy and De Vellis, 1980). Cells were plated with Dulbecco's Modified Eagle Medium (DMEM) with 10% FBS. Media was changed after 24 h. At day 5, when glial cultures were ~80% confluency, they further purified by trypsnizing with 0.05% trypsin for 5 min, centrifugation for 5 min at 1,200 RPM, re-suspending and plating. This is considered passage 1 where residual neuronal cells were eliminated. On day 10, the cells were subjected to passage 2 and collected at day 14 for subsequent analysis.

### Systemic and Stereotactic Administration of Kainic Acid

For systemic administration of KA, P30-45 WT CD-1 or Baf53b x RiboTag mice were used. 20 mg/kg of Kainic Acid (KA) monohydrate (Sigma, K0250) dissolved in PBS was delivered via intraperitoneal injection. Mice were monitored for seizures throughout the time course. Mice were euthanized using isoflourane and decapitated to harvest the brain at indicated time points. For IHC, brain were embedded in Optimal Cutting Temperature (OCT) solution on dry ice and stored in −80°C until further usage. For RNA extraction and immunoprecipitation of RiboTag-mRNA Cortex and hippocampus were dissected out and snap-frozen in liquid nitrogen and stored in −80°C until processing.

Focal administration of KA using sterotactic surgery was performed as described previously (Iijima et al., 2011). Briefly, for anesthetizing WT CD-1 mice (P30–P60), the stock solution of tribromoethanol was made by dissolving 5 g into 5 mL T-amyl alcohol. The working solution was made by diluting to 80-folds into PBS. 0.2 ml working solution per 10 g body weight of mouse was used for anesthesia before mounting the mouse in the stereotax surgery station. A glass micropipette attached to a 10-μL Hamilton syringe was used to deliver KA solution into one hemisphere of the cerebral cortex via a hole made in the occipital bone. Two microliters of 50 μM KA in PBS containing bromphenol blue dye (0.5 mg/ml) was delivered at the rate of 0.4 μl/min. After recovery, mice were monitored for seizures and brain was dissected at indicated time points for RT-PCR, RNA-ISH, and TUNEL assays.

### RNA Extraction

RNA was extracted using Trizol reagent (Thermo Fisher, 15596026) following the manufacturer instructions.

### Semi-quantitative RT-PCR

RNA was quantified using NanoDrop 1000 Spectrophotometer (Thermo Scientific) and equal quantities (total 800–1,000 ng) of RNA was used to synthesize cDNA using PrimeScript High Fidelity RT-PCR Kit (Clonetech, R022A). cDNA was then PCR amplified using following primer pairs.

Nrxn1-SS4-For and Rev (5′-CTGGCCAGTTATCGAACGCT-3′; 5′-GCGATGTTGGCATCGTTCTC-3′), Nrxn2-SS4-For and Rev (5′-CAACGAGAGGTACCCGGC-3′; 5′-TACTAGCCGTAGGTGGCCTT-3′), Nrxn3-SS4-For and Rev (5′-ACACTTCAGGTGGACAACTG-3′; 5′-AGTTGACCTTGGAAGAGACG-3′), Casp3-For and Rev (5′-CTGACTGGAAAGCCGAAACTC-3′; 5′-CGACCCGTCCTTTGAATTTCT-3′), Casp9-For and Rev (5′-TCAGGGGACATGCAGATATGG-3′; 5′-TTGGCAGTCAGGTCGTTCTTC-3′), Cycs-For and Rev (5′-CCAAATCTCCACGGTCTGTTC-3′; 5′-ATCAGGGTATCCTCTCCCCAG-3′), Camk2a-For and Rev (5′-TGGGGACTTGAAAATCTGTGAC-3′; 5′-CACGGGTCTCTTCGGACTG-3′), Syp-For and Rev (5′-AGACATGGACGTGGTGAATCA-3′; 5′-ACTCTCCGTCTTGTTGGCAC-3′), Syn1-For and Rev (5′-CCAATCTGCCGAATGGGTACA-3′; 5′-GCGTTAGACAGCGACGAGAA-3′), Actb-For and Rev (5′-TTGTTACCAACTGGGACGACA-3′; 5′-TCGAAGTCTAGAGCAACATAGC-3′), Arc-For and Rev (5′-AAGTGCCGAGCTGAGATGC-3′; 5′-ACTTCTTCCAGCGCTGTGAG-3′), cFos-For and Rev (5′-CGGGTTTCAACGCCGACTA-3′; 5′-TTGGCACTAGAGACGGACAGA-3′).

PCR amplicons were separated on agrose gel with GelRed gel dye (Biotium) and imaged using the ChemiDoc Gel Imaging station (Bio-Rad). Band intensities were quantified using Image Lab (Bio-Rad) software. To negate the intensity differences that may arise from increased dye incorporation with amplicon size, the band intensity values were normalized to the size of amplicons when the splicing ratio was calculated. Relative expression levels of neurexins in RT-PCR was calculated by combining the intensity of SS4+ and SS4– bands and normalized to Actb.

### Quantitative RT-PCR

For qRT-PCR, equal concentration of RNA was combined with TaqMan Fast Virus 1-Step Master Mix (Life Technologies) and PrimeTime qPCR Probe Assays (Integrated DNA Technologies). The reactions were performed using the QuantStudio 3 Real Time-PCR System (Applied Biosystems). Following probe sets were used; NeuN or Rbfox3 (Mm.PT.58.11398454), vGlut1 (Mm.PT.58.12116555) Aqp4 (Mm.PT.58.9080805), Mbp (Mm.PT.58.28532164), P2ry12 (Mm.PT.58.43542033), Syn1 (Mm.PT.58.32922616), Syp (Mm.PT.58.29275406), Camk2a (Mm.PT.58.8246010), Tubb3 (Mm.PT.58.32393592), Actb (Mm.PT.51.14022423).

### Single Molecule RNA Fluorescent *in-situ* Hybridization (smRNA-FISH)

Focal KA-injected mice were euthanized with isoflurane and decapitated. Brains were quickly removed and embedded in OCT solution on dry ice. Brain tissue was sectioned at 12-μm thickness using a Leica cryostat (CM3050-S) and mounted directly onto Superfrost Plus histological slides and stored at −80°C until further use. Single-molecule RNA-FISH for Nrxn1, Nrxn2, Nrxn3, and Fos mRNA (Advanced Cell Diagnostics, probe cat# 461511-C3, 533531-C2, 505431, and 316921) was performed using the multiplex RNAscope platform (Advanced Cell Diagnostics, 323100) according to the manufacturer instructions for fresh-frozen sections. Samples were mounted using Prolong Gold antifade mounting medium (ThermoFisher, Cat# P36930).

### Immunohistochemistry (IHC)

Mouse brain section and slide were prepared as described in above section. After removing slides from −80°C, tissue were let thaw at RT for 5 min. Tissue were then fixed using ice cold 4% PFA for 30 min at RT. Tissue were washed three times with PBS and incubated with blocking solution (5% normal goat serum and 0.3% TritonX-100 in PBS) for 1 h at RT. Tissue sections were then incubated with primary antibodies diluted in blocking solution overnight at 4°C. Next day, sections were washed three times with PBS and incubated with secondary antibodies (Alexa Fluor conjugated, 1:1,000) diluted in blocking solution for 2 h at RT. Sections were washed three times with PBS and once with water. After drying, sections were mounted using DAPI Fluormount-G (Southern Biotech). Rabbit anti-cFos polyclonal antibody (Millipore, PC05; 1:500) was used in IHC sections.

### Immunocytochemistry (ICC)

For immunocytochemistry, cells were gently washed with warm PBS and fixed for 10 min at RT with 4% PFA in PBS. Following fixation, cells were washed three times with PBS and permeabilized and blocked for 1 h at RT using 0.3% Triton-X 100 and 5% normal goat serum diluted in PBS (blocking solution). Following this step, cells were incubated with primary antibodies diluted in the blocking solution for overnight shacking at 4°C. Next day, cells were washed three times with PBS and then incubated with secondary antibodies (Alexa Fluor conjugated, 1:1,000) diluted in blocking solution for 1 h at RT. Cells were then washed three times with PBS and the coverslips were mounted on glass microscope slides with DAPI-Fluoromount-G mounting solution (Southern Biotech). Following antibodies and dilutions were used; chicken anti-MAP2 (EnCor Biotechnology, CPCA-MAP2; 1:1,000) and mouse anti-GFAP antibody (Neuromab, 75–240; 1:1,000).

### TUNEL Assay

Tissue sections (10–16 micron thickness) from focal KA-injected brain were used for TUNEL assay. Click-iT Plus TUNEL Assay for *in situ* Apoptosis Detection with Alexa Fluor 488 dye (Thermo Fisher, C10617) kit was used following the manufacturer instruction.

### Immunoprecipitation of RiboTag-mRNA

Immunoprecipitation of polyribosome-bound (RiboTag) mRNA was carried out as described previously (Sanz et al., 2009) with minor modifications. Frozen hippocampus or cortex samples were thawed in fresh homogenization buffer at 10% weight/volume and ground using Dounce homogenizer. Homogenates were then purified by centrifugation and 10% of the supernatant was used as input. The remaining supernatant lysate was incubated with pre-washed anti-HA magnetic beads (Thermo) in a rotating mixer overnight at 4°C. The beads were washed three times with a high-salt buffer and mRNA was eluted with RLT lysis buffer (Qiagen) containing 2-mercaptoethanol. RNA was extracted from input and IP samples using RNeasy Micro kit (Qiagen) and the concentration was measured using NanoDrop spectrophotometer and stored at −80°C until further qPCR and RT-PCR analysis.

### Image Acquisition

RNA-ISH, IHC, ICC, and TUNEL samples were imaged using VS120 automated slide scanner (Olympus). For ICC of neuronal-astroglial co-cultures, whole coverslips were imaged and the mean fluorescence intensity (MFI) was calculated using Image J software.

### RNA-Seq and Differential Splicing Analysis

Publicly available RNA-seq datasets were downloaded from Gene Expression Omnibus (GEO) repository for analysis. Datasets from Ding et al. (2017) were (GEO accession number and sample details): GSM2460426 (Control_rep1_RNA-seq), GSM2460428 (Control_rep2_RNA-seq), GSM2460427 (KCl_rep1_RNA-seq), GSM2460429 (KCl_rep2_RNA-seq). Datasets from (Quesnel-Vallieres et al., 2016) were: GSM2395151 (Untreated neurons replicate 1) GSM2395152 (Untreated neurons replicate 2) GSM2395155 (3 h KCl treatment replicate 1), GSM2395156 (3 h KCl treatment replicate 2). Datasets from Ataman et al. (2016) were: GSM2278965 (Mouse rep 1 cortical neurons 0 h post-KCl stim), GSM2278968 (Mouse rep 2 cortical neurons 0 h post-KCl stim), GSM2278967 (Mouse rep 1 cortical neurons 6 h post-KCl stim), GSM2278970 (Mouse rep 2 cortical neurons 6 h post-KCl stim).

RNA-seq data analysis, gene set enrichment analysis (GSEA) and differential splicing analysis were analyzed by workflows on Basepair software (https://www.basepairtech.com/) with a pipeline that consisted following steps: (a) Alignment of reads to the transcriptome derived from UCSC genome assembly using STAR with default parameters (Dobin et al., 2013). (b) Measuring of read counts for each transcript was performed using feature counts (Liao et al., 2014). (c) Differential gene expression (DE) was determined using DESeq2 pipeline (Love et al., 2014). For pathway analysis, cut off parameters of read count >10, *p* < 0.05 and p-adjusted (FDR, corrected for multiple hypotheses testing) <0.1 were used. Differential splicing analysis was performed using LeafCutter tool by incorporating parameters described in Li et al. (2018).

## Results

### High KCl Depolarization Induces Neuronal Cell Death, Obscuring Neurexin SS4-Alternative Splicing Patterns

**Figure 1:**
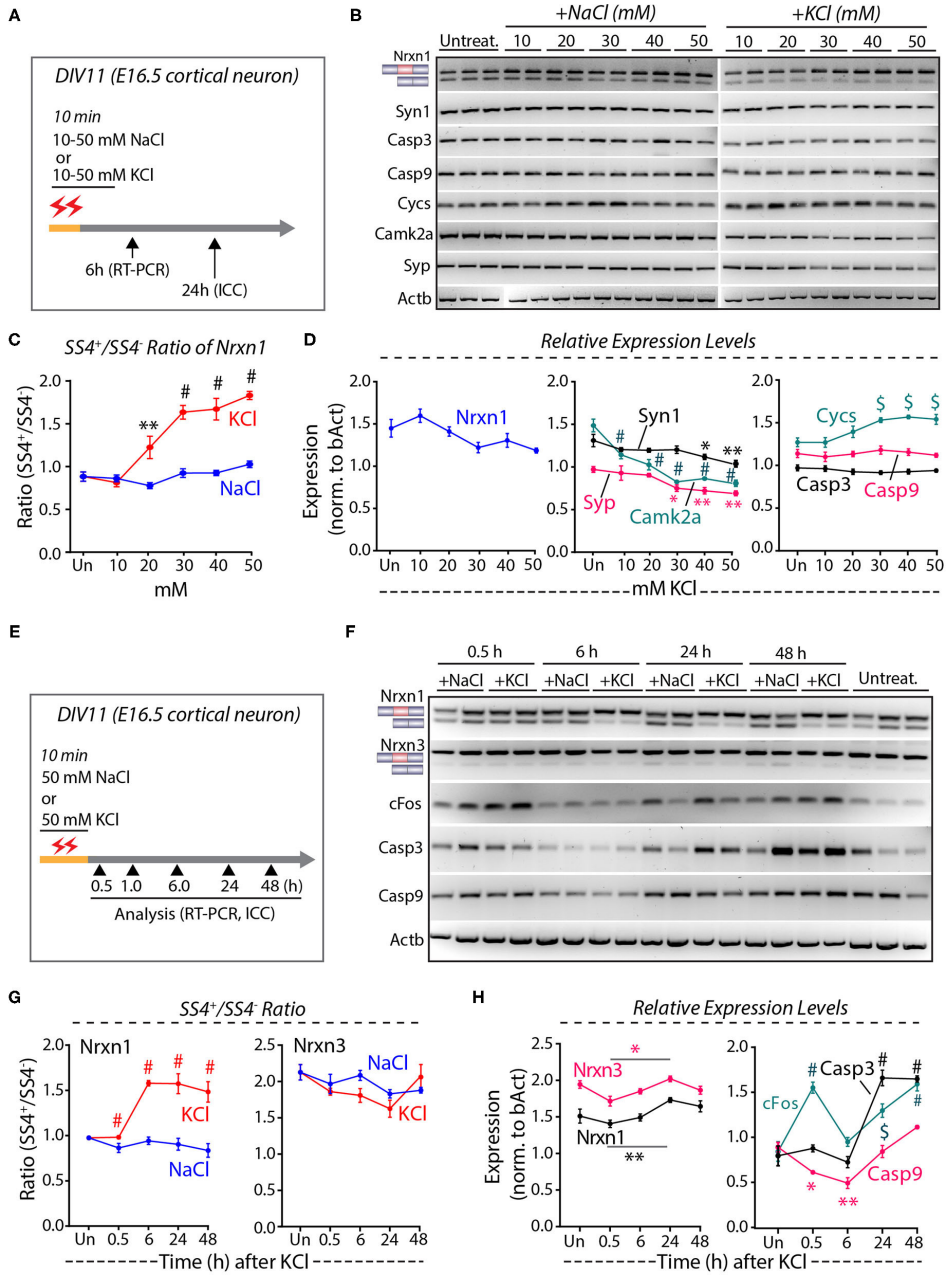
High KCl exposure in cultured cortical neurons leads to reduction in synaptic gene expression and obscures Nrxn1 SS4+/– ratio. **(A)** Experimental paradigm where DIV11 cortical neurons isolated from E16.5 mouse embryos were treated with 10–50 mM NaCl or KCl for 10 min and harvested at 6 and 24 h for RT-PCR and ICC analysis, respectively. **(B)** RT-PCR shows Nrxn1-SS4 alternative splicing and expression of genes encoding key synaptic proteins and cell death markers in untreated and cultures treated with 10–50 mM NaCl or KCl. **(C)** Quantification of Nrxn1 SS4 splicing ratio showing significant difference with increasing KCl concentration compared to NaCl (* = *p* 0.0055, ^#^ = *p* < 0.0001). **(D)** Relative expression levels of total Nrxn1, synaptic markers Syn1, Syp, and Camk2a (note the significant downregulation upon different KCl concentrations), cell death markers Cycs, Casp3, and Casp9 (note the significant upregulation of Cycs upon different KCl concentrations) [Syn1: * = *p* 0.0457, ** = *p* 0.0015; Camk2a: ^#^ *p* < 0.0001; Syp: * = *P* 0.0151, ** = *p* 0.0042, ^*$*^ = *p* 0.0011]; Cys: ^*$*^ = *p* 0.0010 (30 mM), *p* 0.0001 (40 mM) *p* 0.0006 (50 mM). **(E)** Experimental paradigm where DIV11 cortical neurons isolated from E16.5 mouse embryos were treated with 50 mM NaCl or KCl for 10 min and harvested at indicated time points for RT-PCR and ICC analysis. **(F)** RT-PCR analysis shows Nrxn1 and Nrxn3 SS4 alternative splicing and expression of cFos and cell death markers Casp3 and Casp9. **(G)** Quantification of Nrxn1 and Nrxn3 SS4 ratios show significant difference in Nrxn1 SS4 ratio with time duration after KCl treatment compared to NaCl (^#^ = *p* < 0.0001). **(H)** Quantification of relative expression levels of total Nrxn1 and Nrxn3 show increased levels between 0.5 and 24 h (* = *p* 0.0101, ** = *p* 0.0059) and cFos levels show significant induction at 0.5 h and decrease to basal levels at 6 h and increase again at 24 and 48 h. Cell death markers Casp3 is upregulated ta 24 and 48 h whereas Casp9 is downregulated initially at 0.5 and 6 h and upregulated at 24 and 48 h. (cFos: ^#^ = *p* < 0.0001, ^*$*^ = 0.0003; Casp3: ^#^ = *p* < 0.0001; Casp9: *p = 0.0434, **p = 0.0017). All numerical data are represented as means ± SEM. Statistical significance was calculated by two-way ANOVA using Tukey's multiple comparison test.

To quantify the depolarization-induced neurexin SS4 splicing ratio, we exposed cortical cultures derived from E16.5 mouse embryos to 10, 20, 30, 40, and 50 mM KCl or NaCl at DIV11 (days *in-vitro*) for 10 min (Ding et al., 2017). We analyzed the cells by RT-PCR after 6 h and by immunocytochemistry after 24 h (Figure 1A). Semi-quantitative RT-PCR of Nrxn1 SS4 splicing revealed a significant change in the Nrxn1-SS4 splicing ratio (defined as Nrxn1-SS4+/Nrxn1-SS4–), with an increasing preponderance of the SS4+ form associated with increasing KCl concentrations (Figures 1B,C). These data confirm previous results (Ding et al., 2017). Total expression of Nrxn1 and other key synaptic genes (Syn1, Syp, and CamK2a), however, decreased with increasing KCl concentrations, with the suppression of most synaptic gene expression becoming significant at higher KCl concentrations (Figure 1D). Importantly, KCl concentrations above 20 mM also produced a marked increase in the cell death marker Cycs (Boehning et al., 2003), whereas the levels of Casp3 and Casp9 did not change (Figures 1B,D). Addition of NaCl at equivalent concentrations had no effect on any of these parameters.

These results suggest that elevated KCl causes both a change in Nrxn1-SS4 alternative splicing and a possible induction of cell death. To validate these results, we treated the cortical cultures with 50 mM KCl (55 mM final concentration) for 10 min, and harvested mRNAs from the cells at various times between 30 min to 48 h after the treatment (Figure 1E) (Ding et al., 2017). RT-PCR confirmed that K^+^-depolarization induced a significant shift in the Nrxn1-SS4 splicing ratio after 6 h, whereas the equivalent Nrxn3-SS4 splicing ratio was not changed (Figures 1F,G). In parallel with this shift, however, the Nrxn1 and Nrxn3 total mRNA levels exhibited a decrease (Figure 1H). In the same samples, we also measured the mRNA levels of cFos and of Casp3 and Casp9 (Figure 1H). cFos mRNA levels increased transiently at 30 min, and then decreased again to control levels at 6 h as expected. Unexpectedly, however, cFos levels thereafter increased again to high levels at the 24 and 48 h time points (Figure 1H). The second, but not the initial, cFos expression increase was associated with an equivalent large increase in Casp3 and Casp9 expression (Figure 1H). We did not observe such an increase in the experiments of Figure 1D because in that experiment we only examined the 6 h time point (Figure 1H). We next measured the level of neurexin SS4 splicing in neurons that were chronically exposed to 50 mM KCl, and collected RNA at 0.5, 1.0, and 1.5 h time points (Supplementary Figure 1A). We found no significant difference in the SS4 ratio or total neurexin levels (Supplementary Figures 1B–D). Viewed together, these experiments suggest that the K^+^-depolarization induced a biphasic response in cortical cultures: An initial phase of immediate-early gene expression (e.g., cFos) that is not accompanied by a change in Nrxn1-SS4 alternative splicing or increase in cell death, and a later phase of cell death that also features increased cFos expression and produces an apparent change in Nrxn1-SS4 alternative splicing.

**Figure 2:**
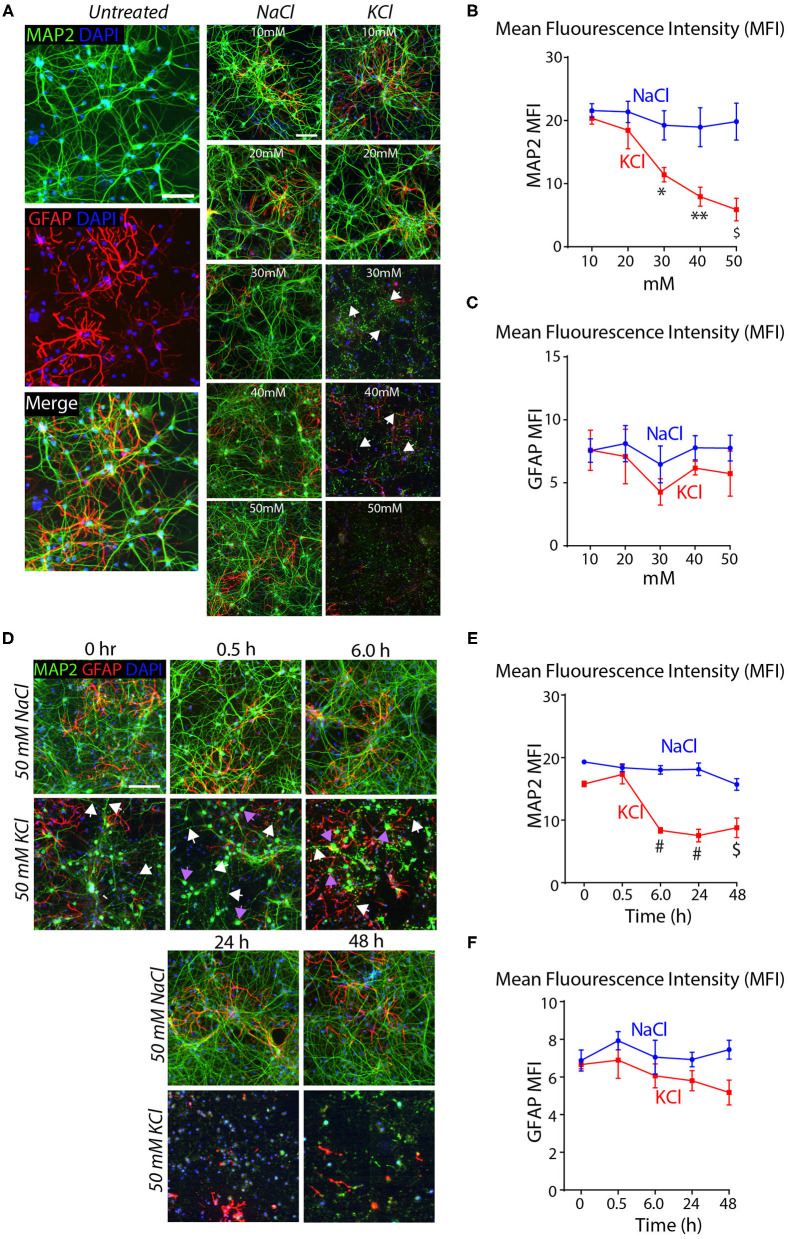
High KCl exposure in cultured cortical neurons leads to reduction in MAP2 protein expression marked by dendritic breakage followed by neuronal death**. (A)** Representative microscopic images of NaCl and KCl treated neurons at different concentrations show remarkable reduction in MAP2 levels from 30 mM KCl and almost no signal at 50 mM. Arrows indicate dendritic breakage. GFAP levels remains the stable across all concentration of KCl and NaCl. Untreated cells shown for comparison. **(B,C)** Quantification of mean fluorescence intensity (MFI) of MAP2 and GFAP immunofluorescence show gradual and significant reduction in MAP2 levels from 30 to 50 mM to KCl compared to NaCl treated cells. (* = *p* 0.0225, ** = *p* 0.0016, ^*$*^ = *p* 0.0004) **(B)**. GFAP levels show no significant difference across various NaCl and KCl concentrations **(C)**. **(D)** Representative microscopic images of 50 mM NaCl and KCl treated neurons collected at different time points show notable staining pattern and reduction in MAP2 levels from as early as 0 h and almost no signal at 48 h. White arrows indicate dendritic breakage and purple arrow indicate mislocalization of MAP2 signals in the cell body. **(E,F)** MFI quantifications show significant reduction in MAP2 immunofluorescence from 6 to 48 h in KCl treated cultures compared to NaCl and no major difference in GFAP levels in same samples (^#^ = *p* < 0.0001, ^*$*^ = *p* 0.0001). All numerical data are represented as means ± SEM. Statistical significance was calculated by two-way ANOVA using Dunnett's multiple comparison test. Scale bars 100 μm.

To test this conclusion, we treated cortical cultures for 10 min with increasing concentrations of KCl and analyzed them by immunocytochemistry for the dendritic marker MAP2 and the astrocyte marker GFAP. Transient exposures (10 min) of cortical cultures to 30–50 mM KCl decreased MAP2 staining (~40–75% decline), but had no significant effect on GFAP levels (Figures 2A–C). The K^+^-depolarization strikingly altered the neuronal cell morphology, with a degeneration of dendrites and swelling of cell bodies (Figure 2A). We then treated cortical cultures with 50 mM KCl for 10 min, and measured the MAP2 and GFAP signals at 30 min to 48 h afterwards as described in Figure 1E. K^+^-depolarization caused a large decrease (~50%) in MAP2 staining after 6 h that persisted for the remaining time of the experiment. Although we also observed a lesser gradual decrease in the GFAP signal after K^+^-depolarization (~20% after 6 h), this change was not significant. Again, neurons exhibited large morphological changes, with fragmented dendrites lacking MAP2 in chronic KCl exposure, apparent from the 0.5 h timepoint onwards (Supplementary Figures 1E–G). In all experiments, we used treatments of cortical cultures with NaCl at equivalent concentrations as a negative control, confirming specificity and ruling out osmotic artifacts (Figure 2). Viewed together, these data indicate that the K^+^-depolarization both causes neuronal cell death and induces a shift in Nrxn1-SS4 alternative splicing, suggesting a causal relationship. Thus, we set out to explore the neuronal cell death might create the impression of a shift in neurexin SS4 alternative splicing.

### Distinct Alternative Splicing in Neurons and Astrocytes Accounts for the Apparent Activity-Dependence of Nrxn1-SS4 Alternative Splicing

**Figure 3:**
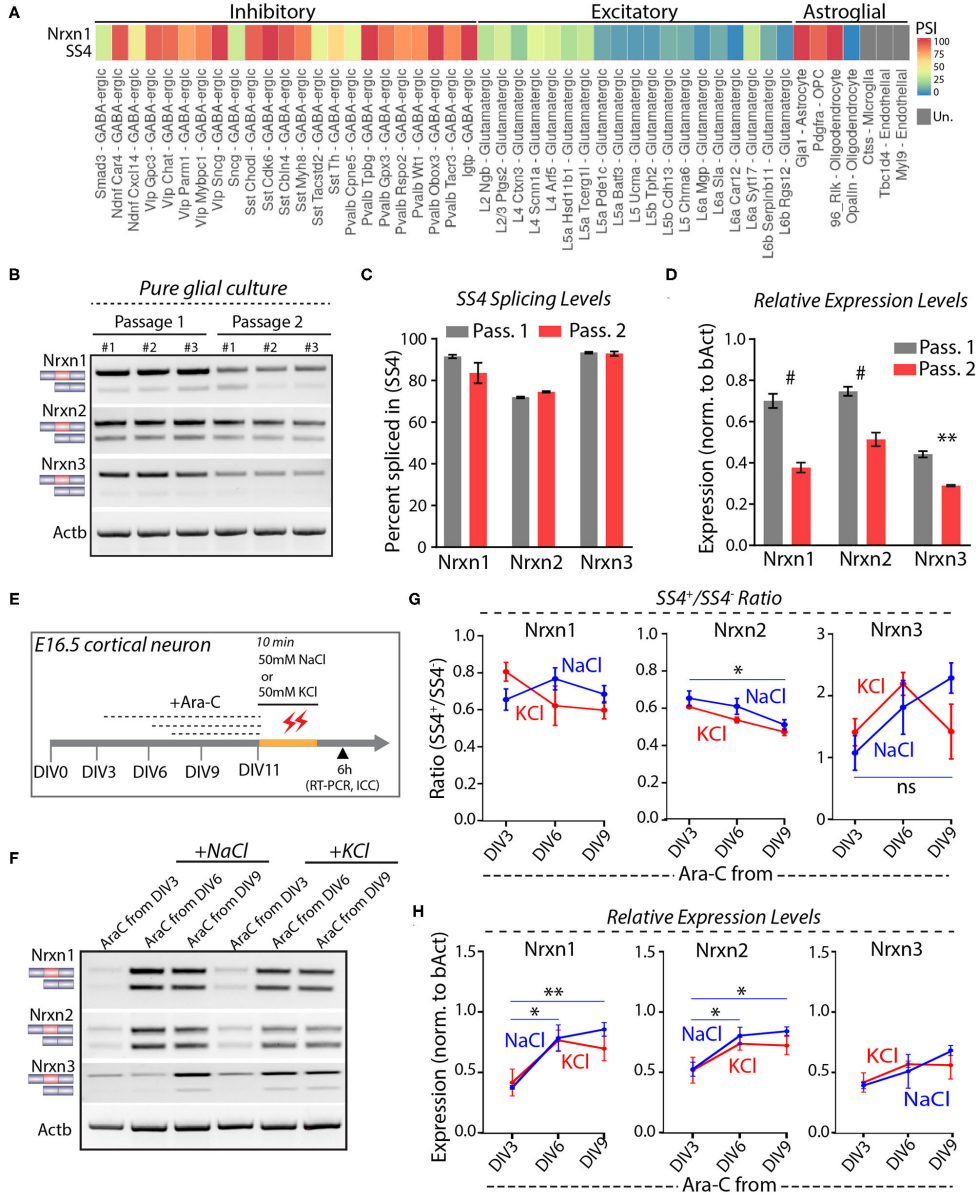
Neuronal and astroglial cells have differential Nrxn SS4 splice pattern**. (A)** ASCOT single cell RNA-seq data (http://ascot.cs.jhu.edu/) from V1 cortex show differential PSI of Nrxn1 SS4 in inhibitory, excitatory neurons, and astroglial cell populations. PSI, Percent Spliced in. Un - undetected. **(B)** RT-PCR shows Nrxn SS4 splice pattern in cells from pure glial culture. Three replicates from two passages shown. **(C)** Quantification of Nrxn SS4 percent spliced in show almost 100% of Nrxn1 and Nrxn3 SS4 spliced-in in glial cells. **(D)** Relative expression levels of all Nrxns in pure glial cells from passage 1 and 2 (^#^ = *p* < 0.0001, ** = *p* 0.0026). **(E)** Experimental paradigm. Cultured cortical neurons isolated from E16.5 mouse embryos were treated with Ara-C from indicated DIV. At DIV11, cells were exposed to high-KCl or NaCl for 10 min and samples were collected after 6 h for RT-PCR and ICC. **(F)** RT-PCR shows Nrxn SS4 splicing in various Ara-C treatment regimens with NaCl and KCl treatments. **(G)** Quantification of splicing ratio shows dynamic change in Nrxn2 and Nrxn3 SS4 splicing between early and late AraC treated control (NaCl) groups. No change in splicing ratio between NaCl and KCl treated cells within specific Ara-C treatment regimens (* = *p* 0.0357, ns = not significant). **(H)** Quantification of relative expression show dynamics of Nrxn1 and Nrxn2, but not Nrxn3 between early and late Ara-C treated control (NaCl) groups. No change in expression levels between NaCl and KCl treated cells within specific Ara-C treatment regimens (* = *p* 0.0153, ** = *p* 0.0054, Nrxn1; * = *p* 0.039, DIV3 vs. DIV6; *p* 0.0204, DIV3 vs. DIV9). All numerical data are represented as means ± SEM. Statistical significance was calculated by two-way ANOVA using Sidak's multiple comparison test.

Astrocytes express high levels of Nrxn1 similar to neurons, but astrocytic Nrxn1 is primarily present as Nrxn1-SS4+ whereas neuronal Nrxn1 is a mixture of Nrxn1-SS4+ and Nrxn1-SS4– (Trotter et al., 2020). Examination of the Alternative Splicing and Gene Expression Summaries of Public RNAseq Data [ASCOT; http://ascot.cs.jhu.edu/ (Ling et al., 2020)] showed that Nrxn1-SS4 is differentially spliced between neurons and astroglial cells. Specifically, the percentage Nrxn1-SS4+ in most GABAergic neurons ranged from 75 to 100% and in most glutamatergic neurons ranged from 0 to 50%, whereas Gja+ astrocytes, Pdgfra+ oligodendrocyte precursor cells (OPC), and Rlk+ oligodendrocytes exhibited almost 100% Nrxn1-SS4+ expression (Figure 3A). We confirmed these data by culturing pure glia from E16.5 embryos over two passages to exclude neurons. Glial cell expressed almost exclusively Nrxn1-, Nrxn2-, and Nrx3-SS4+ splice forms in both passages, whereas total Nrxn1, Nrxn2, and Nrxn3 levels in glial cells declined in the 2^nd^ passage (Figures 3B–D). Thus, the apparent shift in Nrxn1-SS4 alternative splicing induced by K^+^-depolarization could be a result of the survival of astrocytes expressing Nrxn1-SS4+, whereas neurons expressing Nrxn1-SS4– die.

To further test this hypothesis, we treated cortical cultures with cytosine arabinoside (Ara-C), which blocks astrocyte proliferation (Price and Brewer, 2001). Ara-C was added to the cultures at DIV3, DIV6, or DIV9, and cells were exposed to 50 mM KCl or NaCl at DIV11 for 10 min (Figure 3E). RT-PCR analyses, performed 6 h afterwards, showed that AraC prevented the apparent shift in Nrxn1-SS4 alternative splicing (Figures 3F,G). Immunocytochemistry of the cultures revealed that AraC, when given to the cultures earlier (at DIV3 or DIV6), impaired dendritic development, decreased overall GFAP expression, and occluded the cytotoxic effect of high KCl on neurons (Supplementary Figures 2A,B). When AraC was given late (at DIV9), it no longer abolished the cytotoxic effect of KCl on neurons as assessed by MAP2 staining (Supplementary Figure 2C). These results indicate that when K^+^-depolarization induces neuronal cell death in cortical cultures, the persistent expression of astroglial Nrxn1-SS4+ produces the impression of an activity-dependent shift in Nrxn1-SS4 alternative splicing even though Nrxn1-SS4 alternative splicing does not actually change.

### RNAseq Analyses Confirm Expression of Cell-Death Markers Induced by K^+^-Depolarization

**Figure 4:**
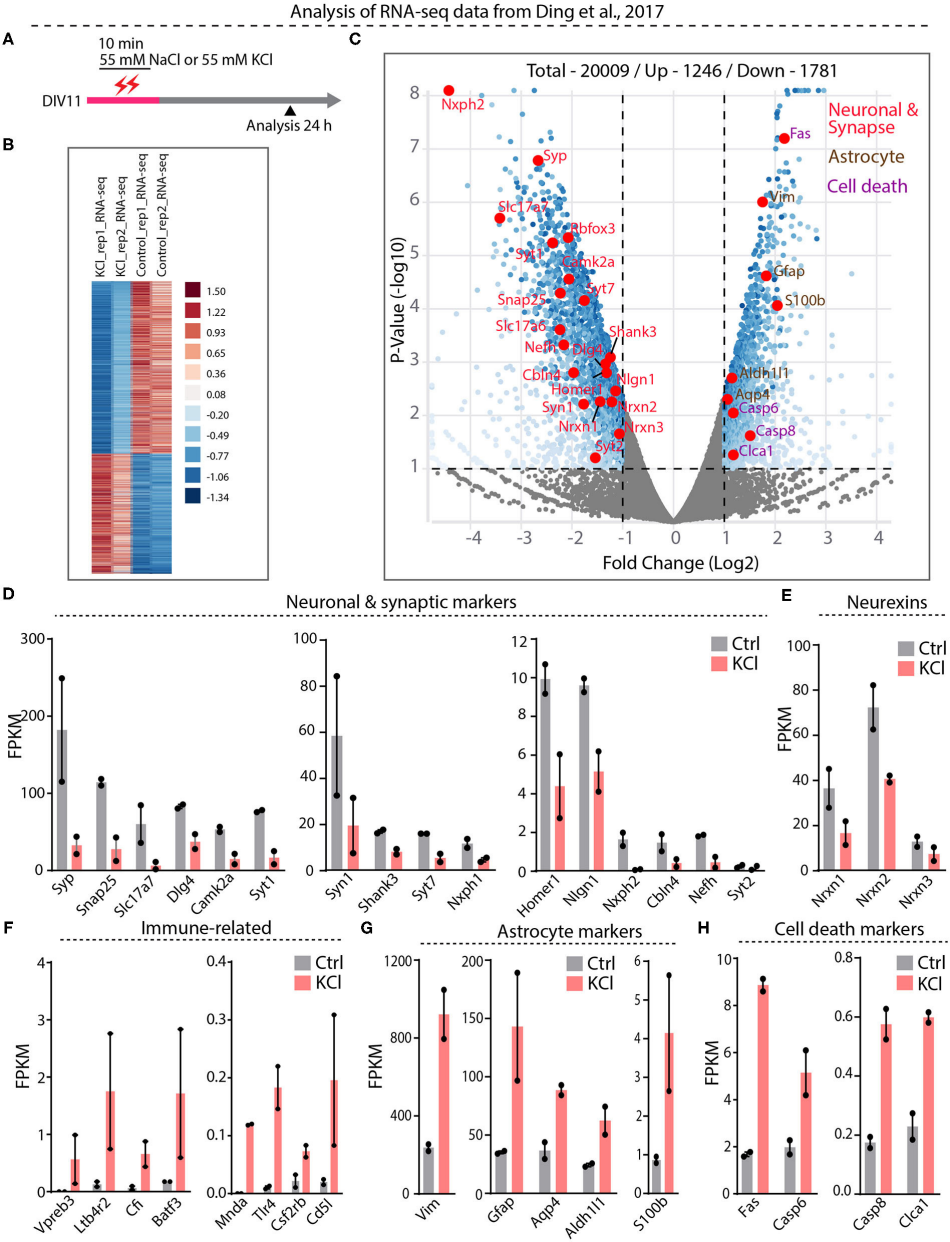
RNA-seq analysis from Ding et al. datasets show significant downregulation of neuronal and synapse markers and upregulation of cell death markers**. (A)** Experimental paradigm used in Ding et al. study, where 55 mM KCl or NaCl was treated on DIV11 for 10 min and samples collected at 24 h for RNA-seq analysis. **(B)** Heatmap show differential gene expression between duplicate KCl and control samples **(C)** Valcano plot showing Log2 fold change of up and down regulated genes. Key synaptic and neuronal markers highlighted in red, enrichment of astrocyte markers in brown and cell death markers in purple. **(D–H)** Individual FPKM-values for neuronal and synaptic markers show down regulation in KCl treated compared to control groups **(D)**, down regulation of all Nrxns **(E)**, up regulation of immune-related genes **(F)**, enrichment of astrocyte markers **(G)**, and upregulation of key cell death markers **(H)**.

Our results suggest that the interpretation of previous papers concluding that Nrxn1-SS4 alternative splicing is highly activity-dependent (Iijima et al., 2011; Ding et al., 2017) may have been confounded by neuronal cell death. To further examine this potential confound, we embarked on a direct analysis of the RNAseq results of Ding et al. (2017), who graciously deposited their data in the public domain (https://www.ncbi.nlm.nih.gov/geo/query/acc.cgi?acc=GSE93682). Ding et al. (2017) obtained these RNAseq data from neurons under experimental conditions identical to those used here (see Figure 1E vs. Figure 4A).

Differential gene expression analysis of the RNAseq data revealed 1,781 downregulated and 1,246 upregulated genes (Figure 4B, Supplementary Table 1). Among downregulated genes, neuronal genes were prominent, and synaptic vesicle proteins and proteins involved in synaptic transmission were generally downregulated more than two-fold (Figures 4C,D, Supplementary Figure 3A). Neurexins and their ligands were similarly decreased in expression, with Nrxn1 levels declining two-fold (Figures 4D,E, Supplementary Figure 3A). The majority of upregulated genes, conversely, was related to inflammatory microglial and astrocytic reactions (Figures 4F–H, Supplementary Figure 3B). Expression of caspases and other cell-death-inducing genes was also increased—in the case of *Fas*, eight-fold (Figure 4H, Supplementary Figures 3C,D). Viewed together, this pattern of gene expression changes is diagnostic of a neuronal cell-death scenario with an astroglial inflammatory reaction. These results suggest that in the experiments of Ding et al. (2017), the KCl treatment also induced cell death with a loss of neuronal gene expression, including that of Nrxn1, and an appearance of regulated Nrxn1-SS4 alternative splicing produced by a change in the relative abundance of neuronal vs. astrocytic Nrxn1.

To deepen our analysis of the effect of sustained KCl-mediated depolarization on neuronal transcription, we analyzed two additional RNAseq datasets from recent studies that involved chronic KCl exposure of cultured neurons (Ataman et al., 2016; Quesnel-Vallieres et al., 2016). In the Quesnel-Vallieres et al. (2016) experiments, E16.5 hippocampal cultures were treated with 55 mM KCl for 0.5 and 3 h (Supplementary Figure 4A). Differential gene expression analysis comparing untreated and 3 h post-KCl treatment samples showed significant induction of IEGs such as Fos, Npas4, and Arc (Supplementary Figures 4B–D, Supplementary Table 2). While there was no significant de-enrichment of synaptic or neuronal markers, GSEA showed an enrichment of 49 genes that are ontologically categorized as “Regulation of RNA Splicing” (Supplementary Figure 5A, Supplementary Table 3) but the splicing factors SLM2 and SAM68 that are known to regulate Nrxn alternative splicing are not represented in the gene sets (Iijima et al., 2011; Traunmuller et al., 2016). However, GSEA analysis also revealed 50 genes from the list of 183 genes that are categorized in “Regulation of Neuron Apoptotic Process” (Supplementary Figure 5B, Supplementary Table 4).

In the Ataman et al. (2016) experiments, E16.5 cortical cultures were silenced at DIV14 overnight with 1 μM TTX and 100 μM D-APV, and then depolarized with 55 mM KCl for 6 h with continued suppression of neuronal excitation using TTX and D-APV (Supplementary Figure 4E). This ingenious protocol thus allows analysis of Ca^2+^-dependent gene expression changes under conditions that suppress excitotoxicity. Differential gene expression analysis uncovered an immediate-early gene expression pattern similar to that of Quesnel-Vallieres et al. (2016) (Supplementary Figures 4F–H, Supplementary Table 5). No significant de-enrichment of synaptic or neuronal markers was present, suggesting that no prominent neuronal cell death occurred. Although no enrichment for “Regulation of RNA Splicing” was observed, differential splicing analysis identified 82 genes with regulated splicing events (Supplementary Figure 6B, Supplementary Table 6). However, neurexins were not present among the 82 genes with differentially regulate alternative splicing, suggesting that even in these experiments in which the neurons appear to be healthy, massive neuronal stimulation did not change alternative splicing of neurexins (Supplementary Figures 5B, 6A). In addition, 60 genes among the 183 upregulated genes were categorized as “Regulation of Neuron Apoptotic Process” (Supplementary Figure 6A, Supplementary Table 7), indicating that even with neuronal silencing, extensive stimulation of neurons triggers a pro-apoptotic gene expression program.

### Kainic Acid-Induced Neuronal Activation Does Not Alter Nrxn1-SS4 Splicing *in vivo*

**Figure 5:**
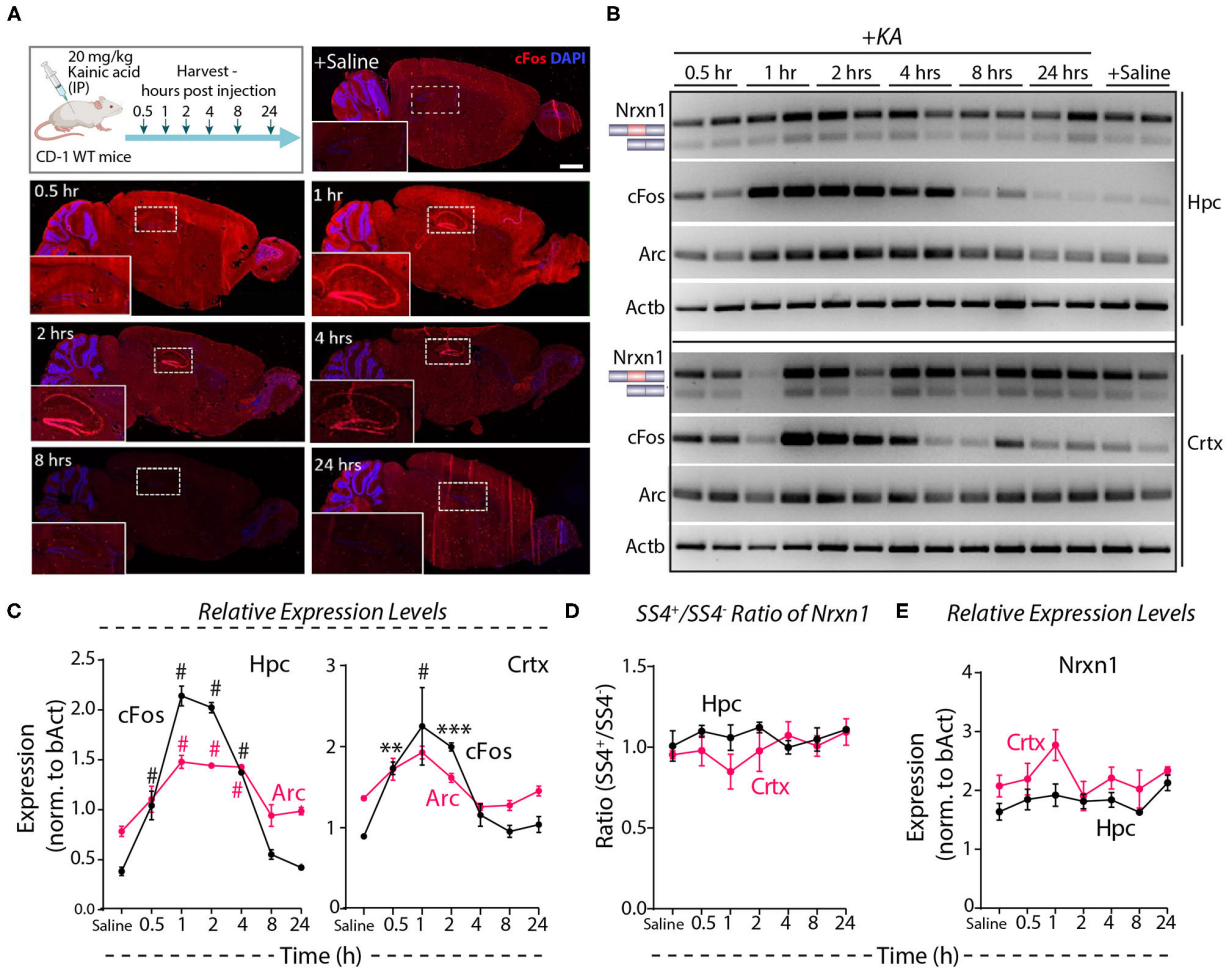
Neuronal activity induced by systemic KA administration does not lead to Nrxn1 SS4 alternative splicing**. (A)** Experimental paradigm where CD-1 WT mice received 20 mg/kg KA and brains were collected at indicated time points. IHC images show induction of cFOS expression in hippocampus (insets) at 1 h and continued expression until 24 h post-injection. **(B)** RT-PCR for Nrxn1 SS4 splicing, cFos, Arc expression in hippocampus (Hpc) and cortex (Crtx) at different time points after KA injection. **(C)** Quantification of relative expression levels of cFos and Arc show significant induction between 0.5 and 4 h in Hpc and 0.5 and 2 h post-KA injection in Crtx (^#^ = *p* < 0.0001, ** = *p* 0.0067, *** = *p* 0.0002). **(D)** Quantification of Nrxn1 SS4+/– ratio shows no significant difference in Hpc and Crtx across all time points post-KA injection. **(E)** Quantification of relative expression levels of total Nrxn1 show no major change across different time points in Crtx and Hpc. All numerical data are represented as means ± SEM. Statistical significance was calculated by two-way ANOVA using Tukey's multiple comparison test. Scale bar 100 μm.

Systemic administration of the glutamate receptor agonist KA induces massive synchronous activation of neurons *in vivo* (Wang et al., 2005). To test the effect of such activation on Nrxn1-SS4 alternative splicing, we injected adult mice with KA (20 mg/kg) intraperitoneally, and analyzed them at 0.5–24 h after injections. Immunohistochemistry showed prominent cFos expression in the hippocampus at the 1, 2, and 4 h time points (Figure 5A). RT-PCR revealed a 2- to 4-fold elevated cFos and Arc expression both in the hippocampus and the cortex between 0.5 and 4 h after injections, with a peak at 1 h after injections, indicating that KA efficiently stimulated neuronal activity throughout the brain (Figures 5B,C). However, the Nrxn1-SS4 splicing ratio was not altered by KA-induced neuronal stimulation *in vivo* in either the hippocampus or the cortex (Figure 5D). Moreover, total Nrxn1 levels did not significantly change (Figure 5E). These results are consistent with a recent study that surveyed alternative exon usage upon systemic administration of KA using RNAseq, and found no evidence for differential neurexin alternative splicing (Denkena et al., 2020).

**Figure 6:**
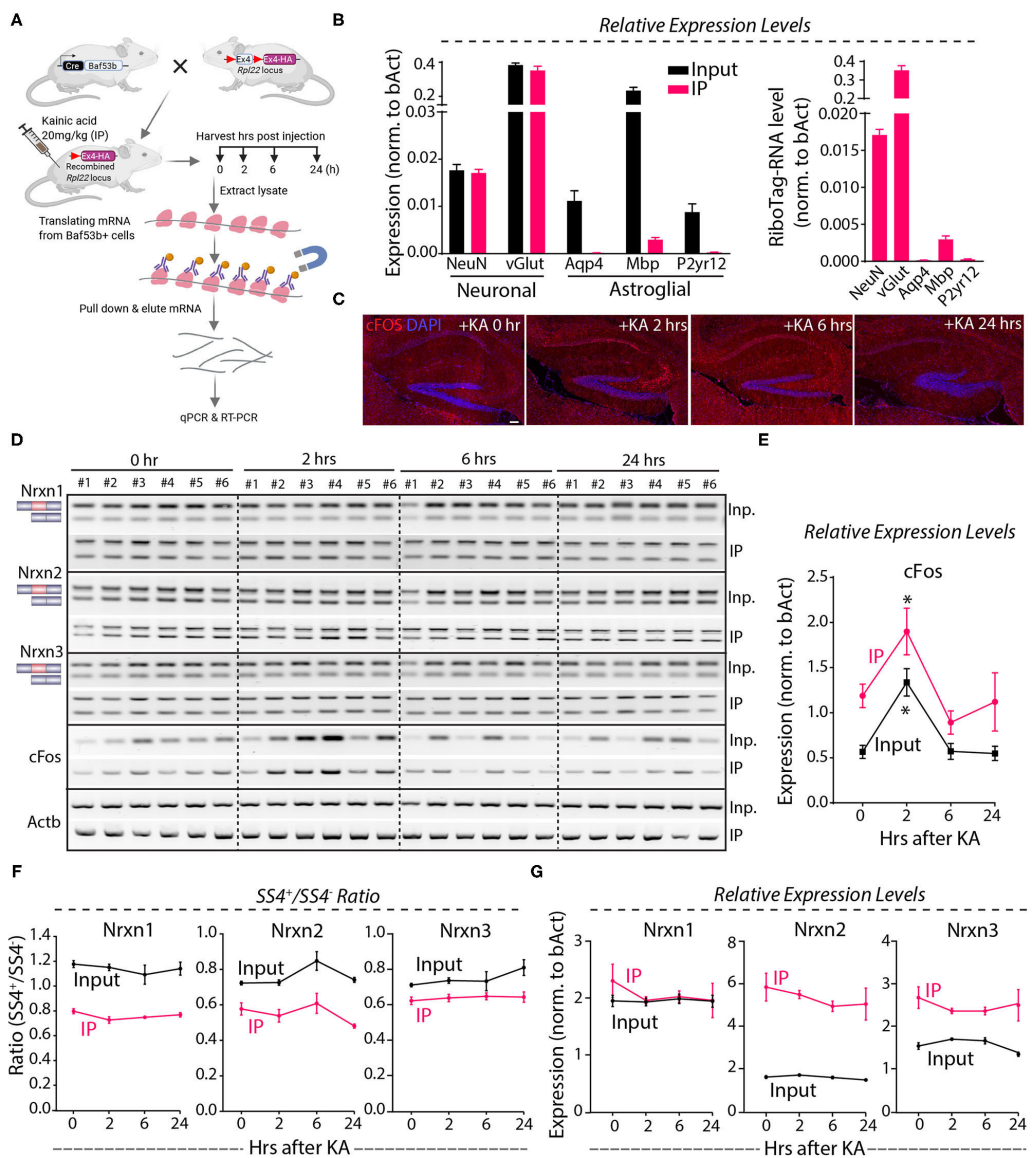
KA-induced activity followed by neuronal specific mRNA enrichment using Baf53b-Cre x RiboTag approach show no change in Nrxn SS4 alternative splicing. **(A)** Baf53b-Cre x RiboTag experimental paradigm. Mice expressing Cre under neuronal-specific Baf53b promotor crossed with RiboTag mice. Adult mice heterozygous for both alleles received 20 mg/kg KA intraperitoneally. Hippocampus harvested at indicated time points. Poly-ribosome bound mRNA isolated using immunoprecipitation (IP) with HA beads and used for subsequent qPCR and RT-PCR. **(B)** Quality control qPCR shows enrichment of neuronal markers and de-enrichment of astroglial markers. **(C)** Microscopic images of IHC in the hippocampus shows KA-induced cFos expression at 2 h post-injection. **(D)** RT-PCR shows Nrxn SS4 splicing and cFos expression in IP and input samples in six mice in each time point. **(E)** Quantification of relative expression of cFos show high level in IP compared to input mRNA. Significant induction of cFos at 2 h in both IP and input samples shown [* = *p* 0.0103 (IP), * = *p* 0.0191 (input)]. **(F)** Quantification of Nrxn SS4 splicing ratio shows marked difference between IP and input mRNA but no significant difference within IP or input samples across different time points **(G)** Quantification of Nrxn relative expression levels show general difference in IP and input mRNA, except for Nrxn1 but no significant difference with in IP or input samples across different time points. All numerical data are represented as means ± SEM. Statistical significance was calculated by two-way ANOVA using Sidaks's multiple comparison test. Scale bar 100 μm.

Because the Nrxn1-SS4 splice pattern varies between neurons and astroglial cells (Figures 3A–C), it is possible that the lack of a KA-induced change in the Nrxn1-SS4 splice ratio was occluded by differential changes in neurons and astrocytes. To address this possibility, we employed a neuron-specific genetic RiboTag strategy to isolate translating neuronal mRNAs (Figure 6A). We injected KA into Baf53b-Cre mice [which exhibit pan-neuronal Cre expression (Zhan et al., 2015) that were crossed with RiboTag mice (Sanz et al., 2009)]. We then harvested the hippocampus at 2–24 h after injections, immunoprecipitated polyribosome-bound mRNAs, and analyzed them by qRT-PCR. Quality control measurements showed an enrichment for neuronal genes and a loss of glial markers in the isolated mRNA (Figure 6B). Immunohistochemistry revealed induction of cFos at 2 h after the KA administration that correlated with mRNA levels (Figures 6C–E), demonstrating that the KA injection stimulated neurons. However, measurements of the SS4 splicing ratio of all three neurexins failed to uncover any stimulation-induced changes in both the neuronal and the total input mRNA (Figure 6F), indicating that neuronal activity induced by systemic KA administration did not alter neurexin SS4 alternative splicing. Moreover, total neurexin levels were also not changed (Figure 6G). Thus, neuronal activation in the hippocampus by KA does not regulate neurexin SS4 alternative splicing.

### Focal KA Injection Into Cerebellum May Alter Neurexin SS4 Alternative Splicing but Suppresses Neurexin Expression

**Figure 7:**
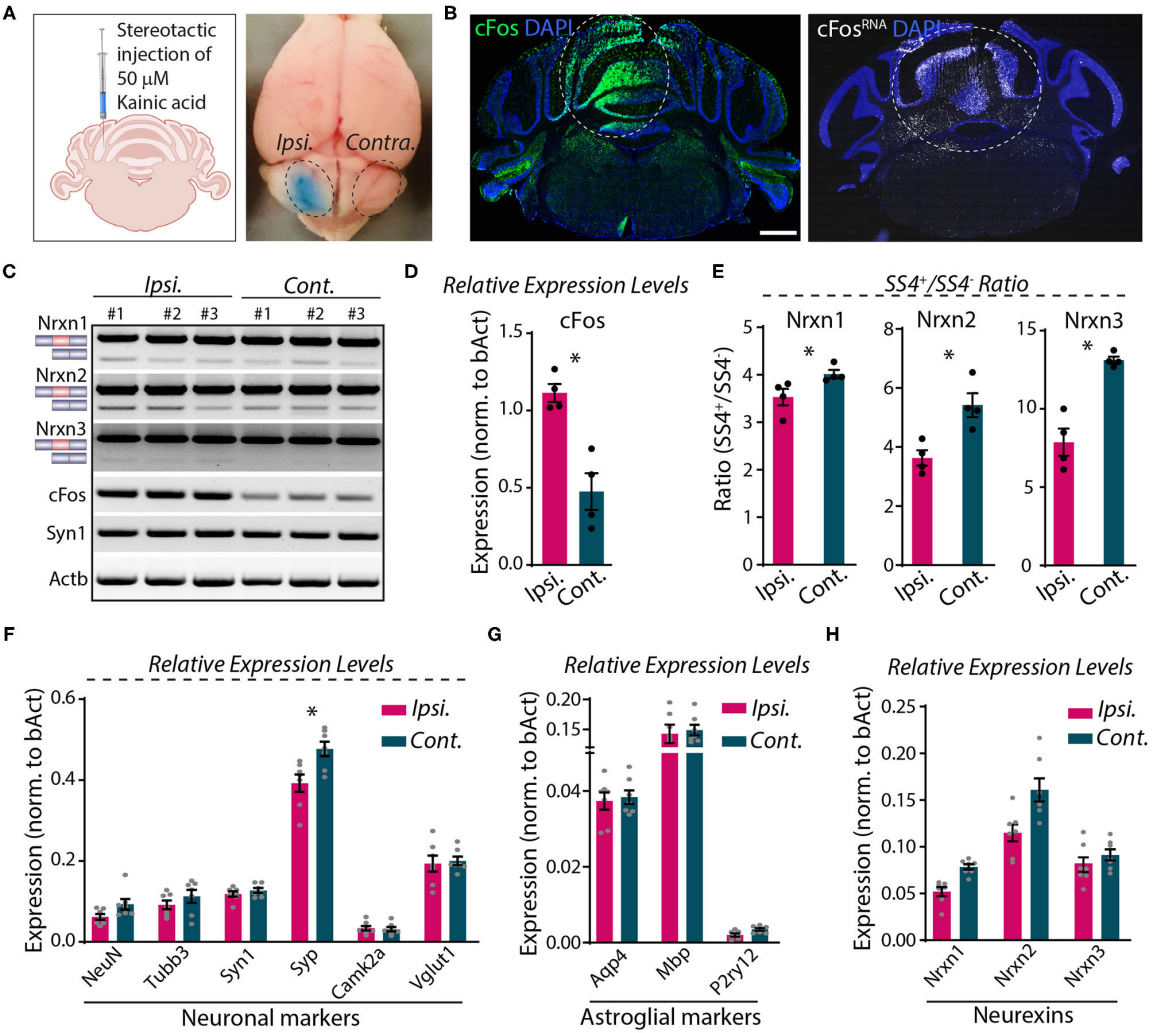
Focal injection of KA in cerebral hemisphere induces Nrxn SS4 alternative splicing**. (A)** Schematic of experimental paradigm. Fifty millimolars of KA stereotactically injected to ipsilateral cerebellum. Dotted area indicates KA-injected area with bromophenol blue dye and uninjected contralateral side. **(B)** Induction of cFos expression at protein (left) and RNA (right) levels in the injected area (dotted lines). **(C)** RT-PCR shows Nrxn SS4 splicing and cFos and Syn1 expression levels in ipsi and contralateral mRNA from three mice. **(D)** Quantification of relative expression levels of cFos shows significant induction in ipsilateral mRNA (* = *p* 0.0286). **(E)** Quantification of Nrxn SS4 splicing ratio shows difference in ipsilateral compared to contralateral side (* = *p* 0.0286). **(F,G)** Quantification of relative expression levels of key neuronal markers show no major change in ipsilateral compared to contralateral side, except a reduction in Syp expression (* = *p* 0.0175). **(F)**. No change in relative expression levels of astroglial markers **(G)**. **(H)** Quantification of relative expression levels of all Nrxns show general reduction in ipsilateral mRNA. All numerical data are represented as means ± SEM. Statistical significance was calculated by Mann-Whitney non-parametric unpaired *t*-test. Scale bar 1 mm.

Direct microinjection of KA into the cerebellum was shown to cause activity-dependent alternative splicing of Nrxn1 at SS4 (Iijima et al., 2011). To explore whether neurexin SS4 alternative splicing might be activity-dependent in the cerebellum, different from the hippocampus or cortex, we stereotactically injected KA into one cerebellar hemisphere (ipsilateral). We examined the injected cerebellum after 5 h, using the uninjected contralateral hemisphere as a control (Figure 7A). Immunohistochemistry, single molecule RNA fluorescent *in-situ* hybridization (smRNA-FISH), and RT-PCR analysis revealed a prominent induction of cFos at the injection site, consistent with KA-induced neuronal activation (Figures 7B–D). Using micro-dissected cerebellar tissue and RT-PCR analyses, we then compared the SS4 splice ratio between the ipsi- and contra-lateral sites for all neurexins. Strikingly, the SS4+/SS4– ratio decreased for all three neurexins. KA caused a relative decrease in the Nrxn1-, Nrxn2-, and Nrxn3-SS4+ levels, which is opposite to what we observed in KCl-treated cells (Figures 7C,E). The changes were most pronounced for Nrxn3 (~40% decrease), whereas they were modest for Nrxn1 (~15% decrease). The expression levels of neuronal and astroglial marker genes, however, did not change except for a small decrease (~15%) in synaptophysin (Syp) (Figures 7F,G). Neurexin mRNA levels were modestly decreased, but this decrease was not statistically significant (Figure 7H).

**Figure 8:**
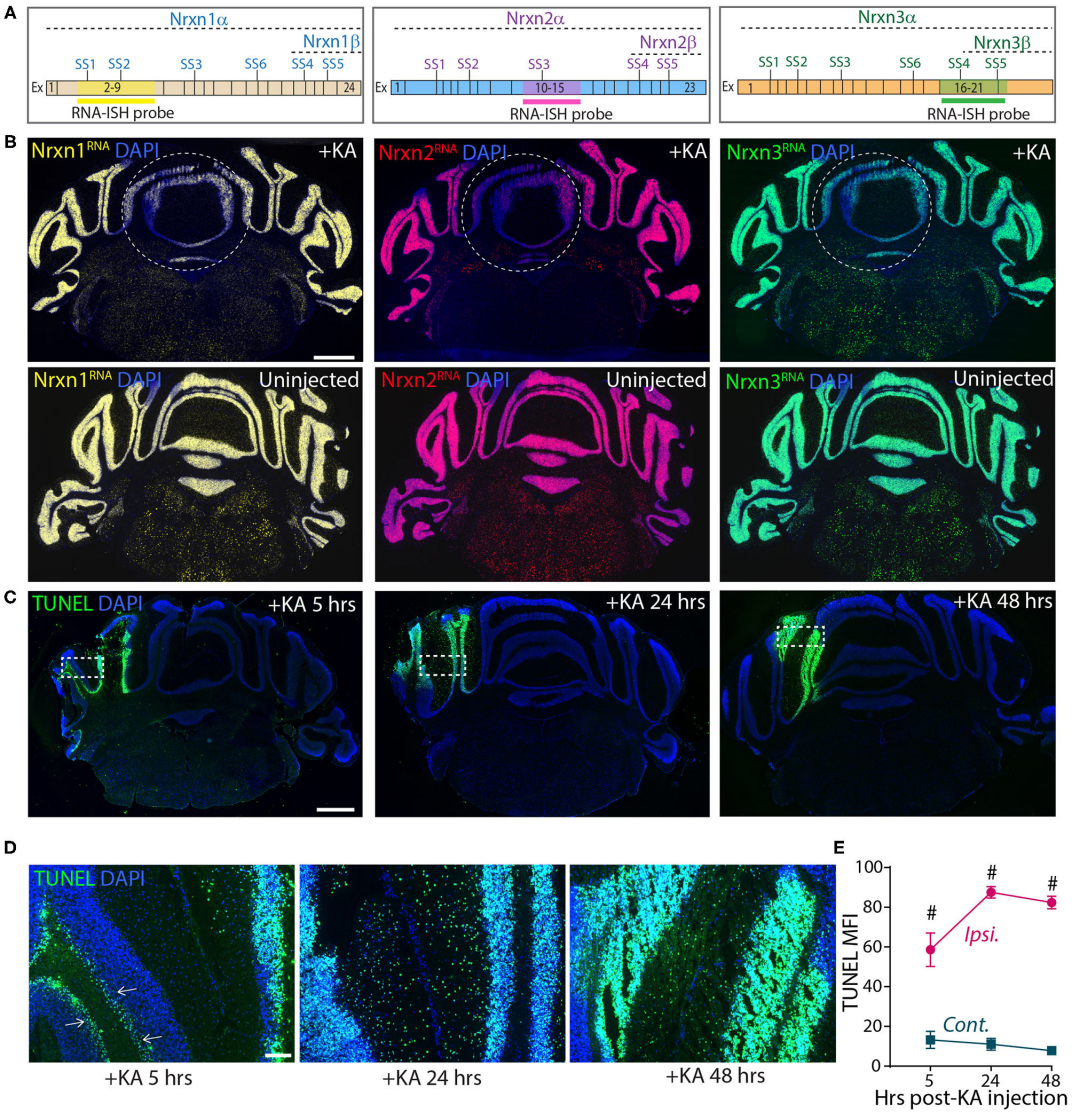
Focal injection of KA in cerebral hemisphere leads to reduced Nrxn expression levels and strong DNA damage. **(A)** Schematic of exon composition of Nrxn-α and -β forms with location of different splice sites and RNA-ISH probes. **(B)** Microscopic images of cerebellar cross sections hybridized with RNA probes against Nrxn1, 2, and 3 shows strong reduction in the expression in KA injected areas. Uninjected controls shown in bottom panel. **(C)** TUNEL labeling of cerebellar cross sections show significant induction of DNA damage in injected areas at 5, 24, and 48 h post-KA injection **(D)** Enlarged images of representative areas (dotted rectangle in **C**) show progression of DNA damage from Purkinji cell layer (arrows) in 5 h to all cells in 48 h post-KA injection. **(E)** Quantification of MFI of TUNEL signal show significant difference in ipsilateral compared to contralateral side. (^#^ = *p* < 0.0001). All numerical data are represented as means ± SEM. Statistical significance was calculated by two-way ANOVA using Sidaks's multiple comparison test. Scale bar 1 mm in B and C, 100 μm in D.

These experiments confirm the results of Iijima et al. (2011), suggesting that KA-induced neuronal activation alters neurexin SS4 alternative splicing. However, they are based on dissecting out injected brain tissue, which could be prone to errors. To more directly analyze the injected area of the cerebellum, we analyzed sections by smRNA-FISH for neurexins (Figure 8). Strikingly, smRNA-ISH uncovered a dramatic loss of Nrxn1, Nrxn2, and Nrxn3 expression in the KA-injected area but not in the surrounding tissue (Figures 8A,B). This result suggests that the dissection of injected tissue was too imprecise to capture the stimulated neurons, and that the KA injection did cause a global change in total neurexin gene expression in the stimulated neurons. To elucidate whether this change was associated with a neurotoxic effect of KA that is known to induce DNA damage and cell death (Kasof et al., 1995; Simonian et al., 1996), we tested for DNA damage at the site of KA exposure. We labeled the KA injection sites 5, 24 and 48 h after treatments using terminal deoxynucleotidyl transferase dUTP nick end labeling (TUNEL) staining. The KA injected sites were strongly positive for TUNEL signals at all-time points (Figures 8C,E), consistent with highly fragmented DNA induced by KA-driven excitotoxicity and apoptosis. At 5 h, TUNEL-positive cells were mostly granule layer cells, whereas at 24 and 48 h, both granule and molecular layer cells were positive for TUNEL signals (Figure 8D). The sustained DNA damage even after 48 h of KA injection indicates long-lasting cell damage that may have a profound impact on the physiology, and confounds the interpretation of the neurexin SS4 alternative splicing observed by RT-PCR.

## Discussion

This study was initiated to explore the mechanistic basis for the activity-dependent alternative splicing of neurexins at SS4, which is of interest because it regulates the glutamate receptor composition of synapses (Aoto et al., 2013; Dai et al., 2019). In pursuing this goal, we first aimed to establish optimal conditions for studying activity-dependent neurexin SS4 alternative splicing, based on pioneering papers that were recently published (Iijima et al., 2011; Ding et al., 2017). However, as we analyzed neurons treated as described in these papers, either in culture or *in vivo*, we noticed that the apparent stimulation-induced change in neurexin SS4 alternative splicing was associated with neuronal cell death. Given the importance of neurexin alternative splicing, we therefore examined in detail the effect of various conditions that are thought to regulate neurexin SS4 alternative splicing on the overall expression of marker genes and on neuronal viability. Our data show that most reported conditions that promote activity-dependent SS4 alternative splicing of neurexins predispose to neuronal cell death, suggesting that the interpretation of such studies is not straightforward. Thus, it is at present unclear whether activity-dependent alternative splicing of neurexins at SS4 is a physiological event.

Application of elevated levels of KCl (55 mM) to cultured neurons has been employed in many studies to trigger activity-dependent gene expression programs and to stimulate alternative splicing of neuronal mRNAs, including neurexins (Martinowich et al., 2003; Greer and Greenberg, 2008; Schor et al., 2009; Kim et al., 2010; Iijima et al., 2011; Rozic et al., 2011, 2013; Ding et al., 2017). This approach is thought to mimic the pattern of gene activation that occurs in response to physiological stimuli in brain (Greer and Greenberg, 2008). However, although elevated extracellular KCl induces membrane depolarization, it may not always actually enhance neuronal activity. Treatment of cultured neurons with 8 mM KCl retains spontaneous neuronal activity, but shifts the pattern of that activity from burst to tonic spike firing (Golbs et al., 2011). Treatment of cultured neurons with KCl above 10 mM conversely diminishes spontaneous activity (Grubb and Burrone, 2010; Rienecker et al., 2020), suggesting that persistent neuronal depolarization does not necessarily induce neuronal stimulation. Furthermore, persistent neuronal depolarization produces sustained increases in intracellular calcium, a key second messenger for numerous cellular processes, including transcription and alternative splicing (Collins et al., 1991; Grubb and Burrone, 2010; Rienecker et al., 2020). In addition, neurons die at concentrations of >50 mm KCl (Collins and Lile, 1989). Na^+^ influx upon KCl-mediated depolarization enhances cell death and induces autophagy (Takahashi et al., 1999; Shehata et al., 2012). In the light of this relationship of KCl to neuronal survival, the utility of the persistent depolarization paradigm as an “activity-inducing system” to study alternative splicing may need re-evaluation. While there are clear merits of using elevated extracellular KCl (extensively reviewed in Rienecker et al., 2020), the inherent confounds and limitations of this approach in studying complex and heterogeneous splice isoforms could outweigh its benefits. The results of our KCl treatment experiments and RNA-seq analyses exemplify and highlight potential disadvantage of this approach.

Historically, the glutamate receptor agonist KA has been used as a neurotoxin to induce cell death to study neurodegeneration in rodents (Pollard et al., 1994; Ankarcrona et al., 1995; Simonian et al., 1996; Wang et al., 2005; Kuzhandaivel et al., 2010). KA is 30-fold more neurotoxic than glutamate and activates kainite-type ionotropic glutamate receptors (Bleakman and Lodge, 1998). Kainate receptor activation by KA increases intracellular Ca^2+^, elevates reactive oxygen species, and stimulates multiple other biochemical reactions, resulting in neuronal cell death (Sun et al., 1992; Cheng and Sun, 1994; Gluck et al., 2000; Milatovic et al., 2002). Similar to the KCl-based assays, KA-induced neuronal activity has also been used to study activity-dependent transcription and alternative splicing of neuronal mRNAs (Iijima et al., 2011; Denkena et al., 2020). Systemic administration of KA may have lesser effects on cell death than focal microinjection of KA into brain regions (Le Duigou et al., 2005). However, systemic KA did not lead to any significant change in neurexin SS4 splicing, although the neurons were massively activated as revealed by induction of cFos. While we observed a small shift in the SS4 alternative splicing of neurexins in areas of the cerebellum that were injected with KA, the strong induction of sustained DNA damage and the suppression of neurexin mRNA levels suggests apoptosis-induced cell death. Moreover, in cerebellar granule cells, KA-induced apoptosis correlates with activation of c-Jun, an immediate-early gene (Cheung et al., 1998). Studies of alternative splicing after KA-induced neuronal activity often do not examine neuronal survival despite the fact that KA is a strong neurotoxic agent (Iijima et al., 2011). The correlation of neurexin SS4 splice changes and immediate-early gene activation with significant DNA damage muddles the interpretation of the alternative splicing results.

While it is possible that activity-dependent neurexin alternative splicing at SS4 occurs in a physiological context, this complex question needs to be tackled using assays that truly mimic neuronal activity in order to address the biological significance. It is likely that the use of massive stimulation paradigms or “hammering” approaches to elicit strong gene expression and alternative splicing changes does more harm than good. Moreover, any major conclusion arising from studies that utilize such approaches may call into question the actual mechanistic underpinning of activity-dependent alternative splicing.

## Data Availability Statement

The datasets presented in this study can be found in online repositories. The names of the repository/repositories and accession number(s) can be found in the methods section.

## Ethics Statement

The animal study was reviewed and approved by Administrative Panel on Laboratory Animal Care (APLAC) at Stanford University.

## Author Contributions

KL-A and TCS conceived the study. KL-A performed the experiments. KL-A and TCS analyzed the data and wrote the manuscript. All authors contributed to the article and approved the submitted version.

## Conflict of Interest

The authors declare that the research was conducted in the absence of any commercial or financial relationships that could be construed as a potential conflict of interest.

## Supplementary Material

Supplementary Figure 1 Chronic exposure of high-KCl induces dendritic breakage and doesn't alter Nrxn SS4 splicing. **(A)** Experimental paradigm. DIV 11 cortical neurons isolated from E16.5 mouse embryos were chronically exposed to 50 mM NaCl or KCl and samples were collected during indicated time points. **(B)** RT-PCR of Nrxn SS4 splicing in NaCl and KCl treated samples at indicated time points. **(C,D)** Quantification of SS4 splice ratio **(C)** and relative expression levels **(C)** of Nrxns shows no difference between KCl and NaCl treatments. **(E)** Microscopic images of ICC stained with MAP2 and GFAP antibodies show marked dendritic breakage (arrows) in KCl treated neurons compared to NaCl. GFAP positive astrocytes show intact morphology. **(F,G)** Quantification of MFI show decrease in MAP2 fluorescence **(F)** in KCl treated cells and no difference in GFAP expression levels **(G)** (^*^ = *p* 0.0313, 1.0 h; ^*^ = *p* 0.0208, 1.5 h). All numerical data are represented as means ± SEM. Statistical significance was calculated by two-way ANOVA using Tukey's multiple comparison test. Scale bar 100 μm.

Supplementary Figure 2 **(A)** Microscopic images of ICC stained with MAP2 and GFAP antibodies show marked reduction in dendritic development (arrows) in cells treated with AraC from DIV3. **(B,C)** Quantification of MFI show decrease in MAP2 fluorescence **(B)** in KCl treated cells with late AraC (from DIV9) treatment **(C)**. Note the increase in GFAP expression level with early to late AraC treatment regimens **(B)**. Scale bar 100 μm.

Supplementary Figure 3 **(A)** Heatmaps from Gene Set Enrichment Analysis (GSEA) of Ding et al. datasets show de-enrichment for several genes encoding neuronal cell body membrane and positive regulators of excitatory postsynaptic potential in KCL treated compared to control neurons. **(B)** Enrichment of inflammatory response genes and **(C,D)** postiive regulators of apoptosis and necrosis in KCL treated compared to control neurons.

Supplementary Figure 4 RNA-seq analysis of Quesnel-Vallières et al., and Ataman et al. datasets. **(A)** Experimental paradigm used in Quesnel-Vallières et al. study, where DIV10 hippocampal neurons isolated from E16.5 mouse embryos chronically exposed to 55 mM KCl and samples collected at 3 h for RNA-seq analysis. **(B)** Heatmap show differential gene expression between duplicate KCl and untreated control samples **(C)** Valcano plot showing Log2 fold change of up and down regulated genes. Note significant upregulation of IEGs, Npas4, cFos, and Arc. No de-enrichment for neuronal markers found. There is an onset of upregulation of Fas, a pro-apoptotic gene. **(D)** List of top 20 up and down regulated genes. **(E)** Experimental paradigm used in Ataman et al. study, where DIV15 cortical neurons isolated from E16.5 mouse embryos were silenced using 1 μM TTX + 100 μM D-APV for overnight before chronically exposing to 55 mM KCl or NaCl. Samples collected at 0 and 6 h for RNA-seq analysis. **(F)** Heatmap show differential gene expression between duplicate KCl and 0 h control samples. **(G)** Valcano plot showing Log2 fold change of up and down regulated genes. Note significant upregulation of IEGs, Npas4, Fos, Junb, and Arc. No de-enrichment for neuronal markers found. There is an onset of upregulation of Fas, a pro-apoptotic gene. **(H)** List of top 20 up and down regulated genes.

Supplementary Figure 5 GSEA analysis of Quesnel-Vallières et al., datasets**. (A)** Heatmap from GSEA analysis show enrichment of 49 genes (of 83) in the GO category: regulation of RNA splicing in 3 h KCl treated cells compared to untreated cells. **(B)** GSEA heatmap shows 50 genes (of 183 in the GO category) that are in the regulation of neuron apoptotic process category.

Supplementary Figure 6 Differential splicing and GSEA analysis from Ataman et al., datasets. **(A)** Bar graph shows genes with differential splicing events analyzed from the datasets. Note that Nrxns does not have any differential splicing events. **(B)** Heatmap from GSEA analysis show enrichment of 60 genes (of 183) in the GO category: regulation of neuron apoptotic process in 6 h KCl treated cells compared to 0 h treated cells.

Supplementary Table 1 List of differentially expressed (DE) genes with up and down regulated genes from Ding et al. (2017) datasets.

Supplementary Table 2 List of differentially expressed (DE) genes with up and down regulated genes from Quesnel-Vallieres et al. (2016) datasets.

Supplementary Table 3 List of GSEA genes of GO category: regulation of RNA splicing from Quesnel-Vallieres et al. (2016) datasets.

Supplementary Table 4 List of GSEA genes of GO category: regulation of neuron apoptosis process from Quesnel-Vallieres et al. (2016) datasets.

Supplementary Table 5 List of differentially expressed (DE) genes with up and down regulated genes from Ataman et al. (2016) datasets.

Supplementary Table 6 List of differentially spliced genes (with number of events) from Ataman et al. (2016) datasets.

Supplementary Table 7 List of GSEA genes of GO category: regulation of neuron apoptosis process from Ataman et al. (2016) datasets.

## References

[B1] Ankarcrona M. Dypbukt J. M. Bonfoco E. Zhivotovsky B. Orrenius S. Lipton S. A.. (1995). Glutamate-induced neuronal death: a succession of necrosis or apoptosis depending on mitochondrial function. Neuron 15, 961–973. 10.1016/0896-6273(95)90186-8 7576644

[B2] Aoto J. Martinelli D. C. Malenka R. C. Tabuchi K. Sudhof T. C. (2013). Presynaptic neurexin-3 alternative splicing trans-synaptically controls postsynaptic AMPA receptor trafficking. Cell 154, 75–88. 10.1016/j.cell.2013.05.060 23827676 PMC3756801

[B3] Ataman B. Boulting G. L. Harmin D. A. Yang M. G. Baker-Salisbury M. Yap E. L.. (2016). Evolution of Osteocrin as an activity-regulated factor in the primate brain. Nature 539, 242–247. 10.1038/nature20111 27830782 PMC5499253

[B4] Bleakman D. Lodge D. (1998). Neuropharmacology of AMPA and kainate receptors. Neuropharmacology 37, 1187–1204. 10.1016/S0028-3908(98)00139-7 9849657

[B5] Boehning D. Patterson R. L. Sedaghat L. Glebova N. O. Kurosaki T. Snyder S. H. (2003). Cytochrome c binds to inositol (1,4,5) trisphosphate receptors, amplifying calcium-dependent apoptosis. Nat. Cell Biol. 5, 1051–1061. 10.1038/ncb1063 14608362

[B6] Boucard A. A. Chubykin A. A. Comoletti D. Taylor P. Sudhof T. C. (2005). A splice code for trans-synaptic cell adhesion mediated by binding of neuroligin 1 to α- and β-neurexins. Neuron 48, 229–236. 10.1016/j.neuron.2005.08.026 16242404

[B7] Boucard A. A. Ko J. Sudhof T. C. (2012). High affinity neurexin binding to cell adhesion G-protein-coupled receptor CIRL1/latrophilin-1 produces an intercellular adhesion complex. J. Biol. Chem. 287, 9399–9413. 10.1074/jbc.M111.318659 22262843 PMC3308797

[B8] Cheng Y. Sun A. Y. (1994). Oxidative mechanisms involved in kainate-induced cytotoxicity in cortical neurons. Neurochem. Res. 19, 1557–1564. 10.1007/BF00969006 7877729

[B9] Cheung N. S. Carroll F. Y. Larm J. A. Beart P. M. Giardina S. F. (1998). Kainate-induced apoptosis correlates with c-Jun activation in cultured cerebellar granule cells. J Neurosci. Res. 52, 69–82. 10.1002/(SICI)1097-4547(19980401)52:1andlt;69::AID-JNR7andgt;3.0.CO;2-I 9556030

[B10] Chih B. Gollan L. Scheiffele P. (2006). Alternative splicing controls selective trans-synaptic interactions of the neuroligin-neurexin complex. Neuron 51, 171–178. 10.1016/j.neuron.2006.06.005 16846852

[B11] Collins F. Lile J. D. (1989). The role of dihydropyridine-sensitive voltage-gated calcium channels in potassium-mediated neuronal survival. Brain Res. 502, 99–108. 10.1016/0006-8993(89)90465-4 2479454

[B12] Collins F. Schmidt M. F. Guthrie P. B. Kater S. B. (1991). Sustained increase in intracellular calcium promotes neuronal survival. J. Neurosci. 11, 2582–2587. 10.1523/JNEUROSCI.11-08-02582.1991 1714495 PMC6575517

[B13] Comoletti D. Flynn R. E. Boucard A. A. Demeler B. Schirf V. Shi J.. (2006). Gene selection, alternative splicing, and post-translational processing regulate neuroligin selectivity for beta-neurexins. Biochemistry 45, 12816–12827. 10.1021/bi0614131 17042500

[B14] Dai J. Aoto J. Sudhof T. C. (2019). Alternative splicing of presynaptic neurexins differentially controls postsynaptic NMDA and AMPA receptor responses. Neuron 102, 993.e5–1008.e5. 10.1016/j.neuron.2019.03.032 31005376 PMC6554035

[B15] Denkena J. Zaisser A. Merz B. Klinger B. Kuhl D. Bluthgen N.. (2020). Neuronal activity regulates alternative exon usage. Mol. Brain 13:148. 10.1186/s13041-020-00685-3 33172478 PMC7656758

[B16] Ding X. Liu S. Tian M. Zhang W. Zhu T. Li D.. (2017). Activity-induced histone modifications govern Neurexin-1 mRNA splicing and memory preservation. Nat. Neurosci. 20, 690–699. 10.1038/nn.4536 28346453

[B17] Dobin A. Davis C. A. Schlesinger F. Drenkow J. Zaleski C. Jha S.. (2013). STAR: ultrafast universal RNA-seq aligner. Bioinformatics 29, 15–21. 10.1093/bioinformatics/bts635 23104886 PMC3530905

[B18] Fuccillo M. V. Foldy C. Gokce O. Rothwell P. E. Sun G. L. Malenka R. C.. (2015). Single-cell mRNA profiling reveals cell-type-specific expression of neurexin isoforms. Neuron 87, 326–340. 10.1016/j.neuron.2015.06.028 26182417 PMC4733560

[B19] Furlanis E. Traunmuller L. Fucile G. Scheiffele P. (2019). Landscape of ribosome-engaged transcript isoforms reveals extensive neuronal-cell-class-specific alternative splicing programs. Nat. Neurosci. 22, 1709–1717. 10.1038/s41593-019-0465-5 31451803 PMC6763336

[B20] Gluck M. R. Jayatilleke E. Shaw S. Rowan A. J. Haroutunian V. (2000). CNS oxidative stress associated with the kainic acid rodent model of experimental epilepsy. Epilepsy Res. 39, 63–71. 10.1016/S0920-1211(99)00111-4 10690755

[B21] Golbs A. Nimmervoll B. Sun J. J. Sava I. E. Luhmann H. J. (2011). Control of programmed cell death by distinct electrical activity patterns. Cereb. Cortex 21, 1192–1202. 10.1093/cercor/bhq200 20966045

[B22] Gomez A. M. Traunmüller L. Scheiffele P. (2021). Neurexins: molecular codes for shaping neuronal synapses. Nat. Rev. Neurosci. 22, 137–151. 10.1038/s41583-020-00415-7 33420412 PMC7612283

[B23] Gorecki D. C. Szklarczyk A. Lukasiuk K. Kaczmarek L. Simons J. P. (1999). Differential seizure-induced and developmental changes of neurexin expression. Mol. Cell. Neurosci. 13, 218–227. 10.1006/mcne.1999.0740 10408888

[B24] Greer P. L. Greenberg M. E. (2008). From synapse to nucleus: calcium-dependent gene transcription in the control of synapse development and function. Neuron 59, 846–860. 10.1016/j.neuron.2008.09.002 18817726

[B25] Grubb M. S. Burrone J. (2010). Activity-dependent relocation of the axon initial segment fine-tunes neuronal excitability. Nature 465, 1070–1074. 10.1038/nature09160 20543823 PMC3196626

[B26] Iijima T. Wu K. Witte H. Hanno-Iijima Y. Glatter T. Richard S.. (2011). SAM68 regulates neuronal activity-dependent alternative splicing of neurexin-1. Cell 147, 1601–1614. 10.1016/j.cell.2011.11.028 22196734 PMC3246220

[B27] Kasem E. Kurihara T. Tabuchi K. (2018). Neurexins and neuropsychiatric disorders. Neurosci. Res. 127, 53–60. 10.1016/j.neures.2017.10.012 29221905

[B28] Kasof G. M. Mandelzys A. Maika S. D. Hammer R. E. Curran T. Morgan J. I. (1995). Kainic acid-induced neuronal death is associated with DNA damage and a unique immediate-early gene response in c-fos-lacZ transgenic rats. J. Neurosci. 15, 4238–4249. 10.1523/JNEUROSCI.15-06-04238.1995 7790908 PMC6577729

[B29] Kim T. K. Hemberg M. Gray J. M. Costa A. M. Bear D. M. Wu J.. (2010). Widespread transcription at neuronal activity-regulated enhancers. Nature 465, 182–187. 10.1038/nature09033 20393465 PMC3020079

[B30] Ko J. Fuccillo M. V. Malenka R. C. Sudhof T. C. (2009). LRRTM2 functions as a neurexin ligand in promoting excitatory synapse formation. Neuron 64, 791–798. 10.1016/j.neuron.2009.12.012 20064387 PMC2829314

[B31] Kuzhandaivel A. Nistri A. Mladinic M. (2010). Kainate-mediated excitotoxicity induces neuronal death in the rat spinal cord *in vitro* via a PARP-1 dependent cell death pathway (Parthanatos). Cell. Mol. Neurobiol. 30, 1001–1012. 10.1007/s10571-010-9531-y 20502958 PMC11498824

[B32] Le Duigou C. Wittner L. Danglot L. Miles R. (2005). Effects of focal injection of kainic acid into the mouse hippocampus *in vitro* and *ex vivo*. J. Physiol. Lond. 569, 833–847. 10.1113/jphysiol.2005.094599 16239280 PMC1464260

[B33] Li Y. I. Knowles D. A. Humphrey J. Barbeira A. N. Dickinson S. P. Im H. K.. (2018). Annotation-free quantification of RNA splicing using LeafCutter. Nat. Genet. 50, 151–158. 10.1038/s41588-017-0004-9 29229983 PMC5742080

[B34] Liao Y. Smyth G. K. Shi W. (2014). featureCounts: an efficient general purpose program for assigning sequence reads to genomic features. Bioinformatics 30, 923–930. 10.1093/bioinformatics/btt656 24227677

[B35] Ling J. P. Wilks C. Charles R. Leavey P. J. Ghosh D. Jiang L.. (2020). ASCOT identifies key regulators of neuronal subtype-specific splicing. Nat. Commun. 11:137. 10.1038/s41467-019-14020-5 31919425 PMC6952364

[B36] Love M. I. Huber W. Anders S. (2014). Moderated estimation of fold change and dispersion for RNA-seq data with DESeq2. Genome Biol. 15:550. 10.1186/s13059-014-0550-8 25516281 PMC4302049

[B37] Lukacsovich D. Winterer J. Que L. Luo W. Lukacsovich T. Foldy C. (2019). Single-cell RNA-Seq reveals developmental origins and ontogenetic stability of neurexin alternative splicing profiles. Cell Rep. 27, 3752.e4–3759.e4. 10.1016/j.celrep.2019.05.090 31242409

[B38] Martinowich K. Hattori D. Wu H. Fouse S. He F. Hu Y.. (2003). DNA methylation-related chromatin remodeling in activity-dependent BDNF gene regulation. Science 302, 890–893. 10.1126/science.1090842 14593184

[B39] Matsuda K. Yuzaki M. (2011). Cbln family proteins promote synapse formation by regulating distinct neurexin signaling pathways in various brain regions. Eur. J. Neurosci. 33, 1447–1461. 10.1111/j.1460-9568.2011.07638.x 21410790

[B40] McCarthy K. D. De Vellis J. (1980). Preparation of separate astroglial and oligodendroglial cell cultures from rat cerebral tissue. J. Cell Biol. 85, 890–902. 10.1083/jcb.85.3.890 6248568 PMC2111442

[B41] Milatovic D. Gupta R. C. Dettbarn W. D. (2002). Involvement of nitric oxide in kainic acid-induced excitotoxicity in rat brain. Brain Res. 957, 330–337. 10.1016/S0006-8993(02)03669-7 12445975

[B42] Pollard H. Charriaut-Marlangue C. Cantagrel S. Represa A. Robain O. Moreau J.. (1994). Kainate-induced apoptotic cell death in hippocampal neurons. Neuroscience 63, 7–18. 10.1016/0306-4522(94)90003-5 7898662

[B43] Price P. J. Brewer G. J. (2001). “Serum-free media for neural cell cultures,” in Protocols for Neural Cell Culture, eds S. Fedoroff A. Richardson (Totowa, NJ: Humana Press), 255–264. 10.1385/1-59259-207-4:255

[B44] Quesnel-Vallieres M. Dargaei Z. Irimia M. Gonatopoulos-Pournatzis T. Ip J. Y. Wu M.. (2016). Misregulation of an activity-dependent splicing network as a common mechanism underlying autism spectrum disorders. Mol. Cell 64, 1023–1034. 10.1016/j.molcel.2016.11.033 27984743

[B45] Rienecker K. D. A. Poston R. G. Saha R. N. (2020). Merits and limitations of studying neuronal depolarization-dependent processes using elevated external potassium. ASN Neuro. 12:1759091420974807. 10.1177/1759091420974807 33256465 PMC7711227

[B46] Rozic G. Lupowitz Z. Piontkewitz Y. Zisapel N. (2011). Dynamic changes in neurexins' alternative splicing: role of Rho-associated protein kinases and relevance to memory formation. PLoS ONE 6:e18579. 10.1371/journal.pone.0018579 21533271 PMC3075264

[B47] Rozic G. Lupowitz Z. Zisapel N. (2013). Exonal elements and factors involved in the depolarization-induced alternative splicing of neurexin 2. J. Mol. Neurosci. 50, 221–233. 10.1007/s12031-012-9919-x 23180095 PMC3622022

[B48] Rozic-Kotliroff G. Zisapel N. (2007). Ca^2+^-dependent splicing of neurexin IIα. Biochem. Biophys. Res. Commun. 352, 226–230. 10.1016/j.bbrc.2006.11.008 17107668

[B49] Sanz E. Yang L. Su T. Morris D. R. McKnight G. S. Amieux P. S. (2009). Cell-type-specific isolation of ribosome-associated mRNA from complex tissues. Proc. Natl. Acad. Sci. U.S.A 106, 13939–13944. 10.1073/pnas.0907143106 19666516 PMC2728999

[B50] Schor I. E. Rascovan N. Pelisch F. Allo M. Kornblihtt A. R. (2009). Neuronal cell depolarization induces intragenic chromatin modifications affecting NCAM alternative splicing. Proc. Natl. Acad. Sci. U.S.A. 106, 4325–4330. 10.1073/pnas.0810666106 19251664 PMC2657401

[B51] Shehata M. Matsumura H. Okubo-Suzuki R. Ohkawa N. Inokuchi K. (2012). Neuronal stimulation induces autophagy in hippocampal neurons that is involved in AMPA receptor degradation after chemical long-term depression. J. Neurosci. 32, 10413–10422. 10.1523/JNEUROSCI.4533-11.2012 22836274 PMC6703735

[B52] Siddiqui T. J. Pancaroglu R. Kang Y. Rooyakkers A. Craig A. M. (2010). LRRTMs and neuroligins bind neurexins with a differential code to cooperate in glutamate synapse development. J. Neurosci. 30, 7495–7506. 10.1523/JNEUROSCI.0470-10.2010 20519524 PMC2896269

[B53] Simonian N. A. Getz R. L. Leveque J. C. Konradi C. Coyle J. T. (1996). Kainic acid induces apoptosis in neurons. Neuroscience 75, 1047–1055. 10.1016/0306-4522(96)00326-0 8938740

[B54] Sudhof T. C. (2017). Synaptic neurexin complexes: a molecular code for the logic of neural circuits. Cell 171, 745–769. 10.1016/j.cell.2017.10.024 29100073 PMC5694349

[B55] Sugita S. Saito F. Tang J. Satz J. Campbell K. Sudhof T. C. (2001). A stoichiometric complex of neurexins and dystroglycan in brain. J. Cell Biol. 154, 435–445. 10.1083/jcb.200105003 11470830 PMC2150755

[B56] Sun A. Y. Cheng Y. Sun G. Y. (1992). Kainic acid-induced excitotoxicity in neurons and glial cells. Prog. Brain Res. 94, 271–280. 10.1016/S0079-6123(08)61757-4 1363145

[B57] Tabuchi K. Sudhof T. C. (2002). Structure and evolution of neurexin genes: insight into the mechanism of alternative splicing. Genomics 79, 849–859. 10.1006/geno.2002.6780 12036300

[B58] Takahashi S. Shibata M. Fukuuchi Y. (1999). Role of sodium ion influx in depolarization-induced neuronal cell death by high KCI or veratridine. Eur. J. Pharmacol. 372, 297–304. 10.1016/S0014-2999(99)00208-3 10395025

[B59] Traunmuller L. Gomez A. M. Nguyen T. M. Scheiffele P. (2016). Control of neuronal synapse specification by a highly dedicated alternative splicing program. Science 352, 982–986. 10.1126/science.aaf2397 27174676

[B60] Treutlein B. Gokce O. Quake S. R. Sudhof T. C. (2014). Cartography of neurexin alternative splicing mapped by single-molecule long-read mRNA sequencing. Proc. Natl. Acad. Sci. U.S.A. 111, E1291–E1299. 10.1073/pnas.1403244111 24639501 PMC3977267

[B61] Trotter J. H. Dargaei Z. Wöhr M. Liakath-Ali K. Raju K. Essayan-Perez S.. (2020). Astrocytic neurexin-1 orchestrates functional synapse assembly. bioRxiv [Preprint]. 10.1101/2020.08.21.262097

[B62] Uemura T. Lee S. J. Yasumura M. Takeuchi T. Yoshida T. Ra M.. (2010). Trans-synaptic interaction of GluRδ2 and Neurexin through Cbln1 mediates synapse formation in the cerebellum. Cell 141, 1068–1079. 10.1016/j.cell.2010.04.035 20537373

[B63] Ullrich B. Ushkaryov Y. A. Sudhof T. C. (1995). Cartography of neurexins: more than 1000 isoforms generated by alternative splicing and expressed in distinct subsets of neurons. Neuron 14, 497–507. 10.1016/0896-6273(95)90306-2 7695896

[B64] Wang Q. Yu S. Simonyi A. Sun G. Y. Sun A. Y. (2005). Kainic acid-mediated excitotoxicity as a model for neurodegeneration. Mol. Neurobiol. 31, 3–16. 10.1385/MN:31:1-3:003 15953808

[B65] Zhan X. Cao M. Yoo A. S. Zhang Z. Chen L. Crabtree G. R.. (2015). Generation of BAF53b-Cre transgenic mice with pan-neuronal Cre activities. Genesis 53, 440–448. 10.1002/dvg.22866 26077106 PMC4514543

