# Supplementary figures and images for: The Perils of Navigating Activity-Dependent Alternative Splicing of Neurexins
Source: Front Mol Neurosci. 2021 Mar 9;14:659681. doi: 10.3389/fnmol.2021.659681 (PMC7985251; doi:10.3389/fnmol.2021.659681)

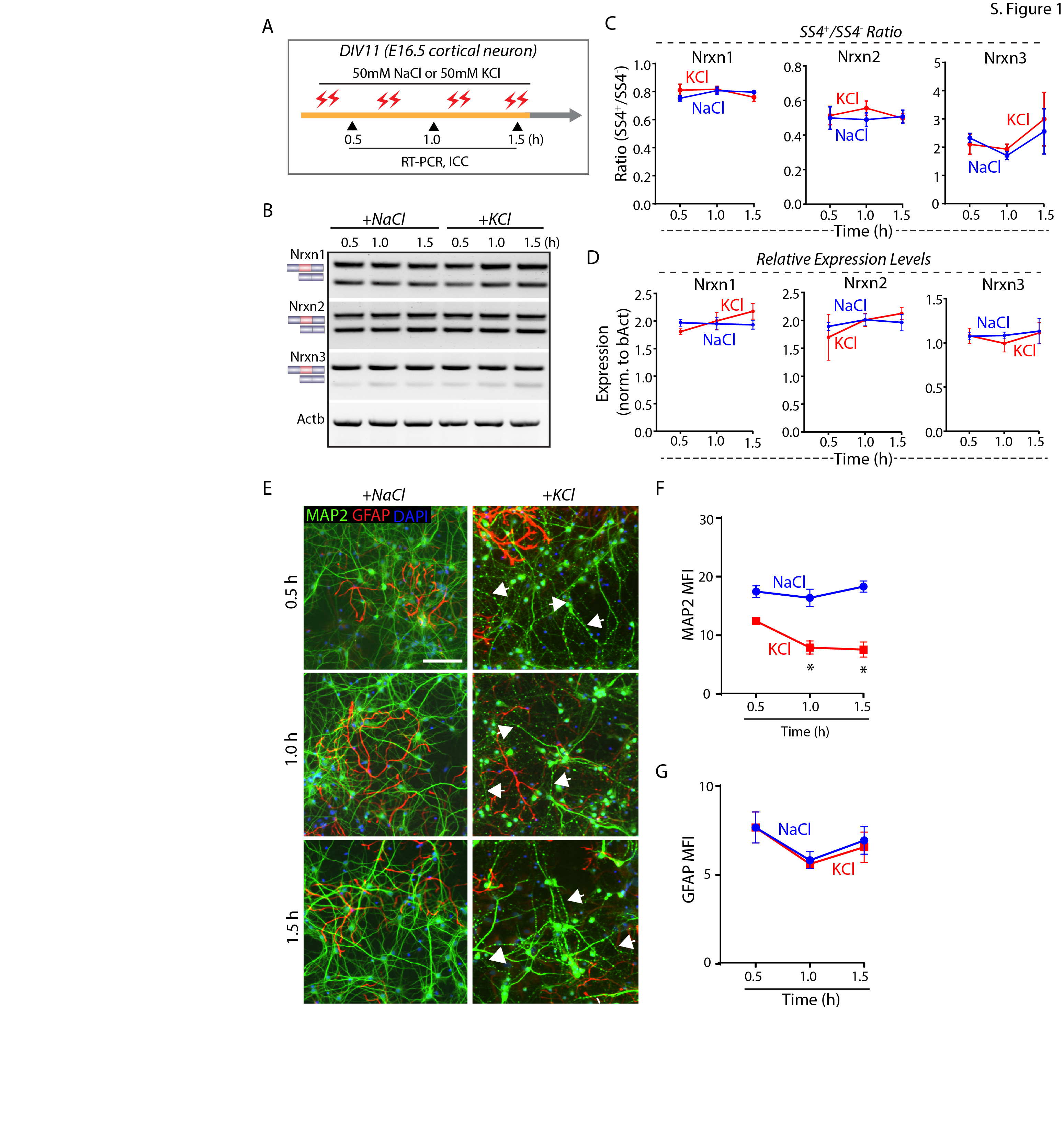

Supplement: Supplementary Figure 1 — Chronic exposure of high-KCl induces dendritic breakage and doesn't alter Nrxn SS4 splicing. (A) Experimental paradigm. DIV 11 cortical neurons isolated from E16.5 mouse embryos were chronically exposed to 50 mM NaCl or KCl and samples were collected during indicated time points. (B) RT-PCR of Nrxn SS4 splicing in NaCl and KCl treated samples at indicated time points. (C,D) Quantification of SS4 splice ratio (C) and relative expression levels (C) of Nrxns shows no difference between KCl and NaCl treatments. (E) Microscopic images of ICC stained with MAP2 and GFAP antibodies show marked dendritic breakage (arrows) in KCl treated neurons compared to NaCl. GFAP positive astrocytes show intact morphology. (F,G) Quantification of MFI show decrease in MAP2 fluorescence (F) in KCl treated cells and no difference in GFAP expression levels (G) (* = p 0.0313, 1.0 h; * = p 0.0208, 1.5 h). All numerical data are represented as means ± SEM. Statistical significance was calculated by two-way ANOVA using Tukey's multiple comparison test. Scale bar 100 μm. [file Image_1.jpeg]

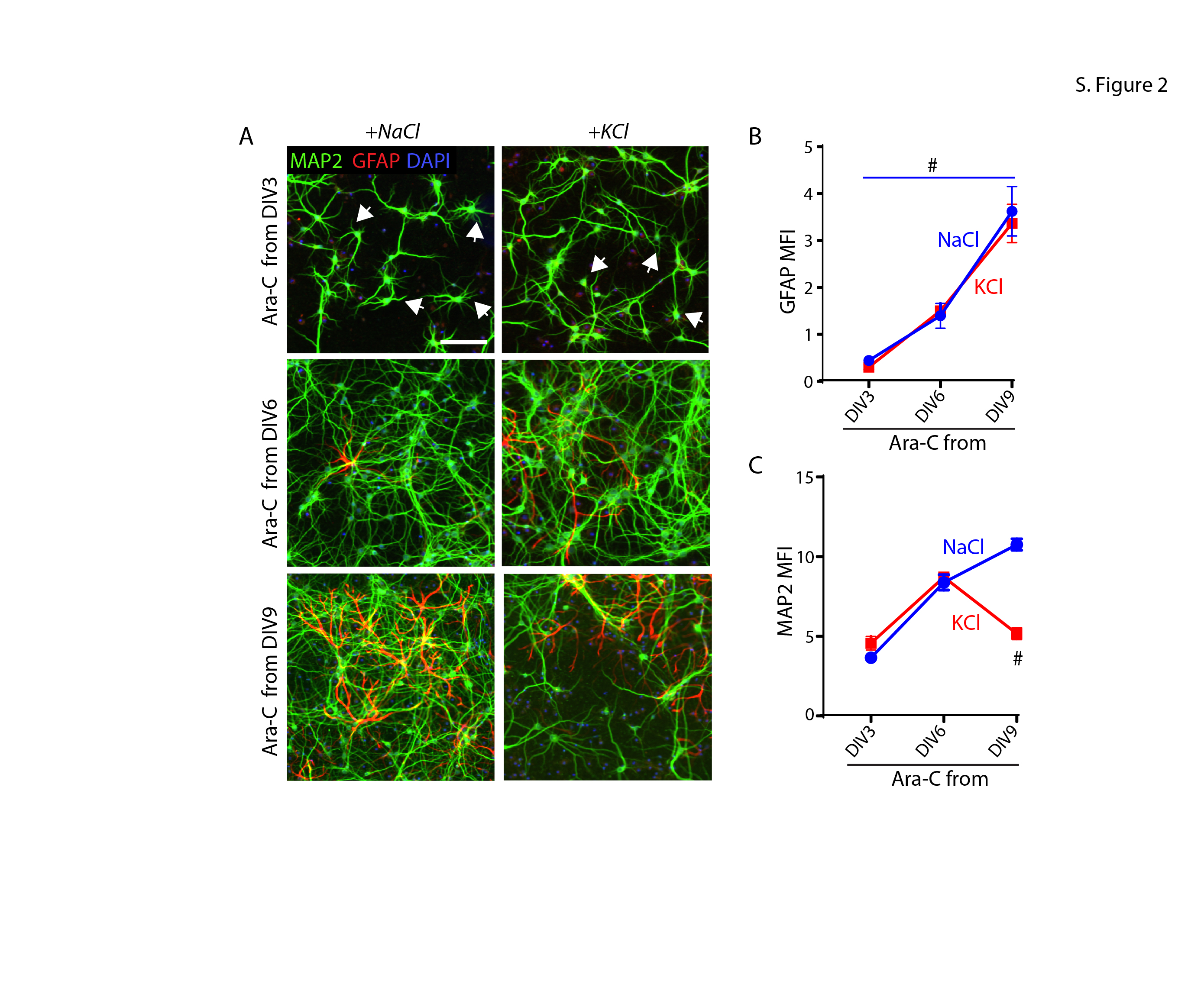

Supplement: Supplementary Figure 2 — (A) Microscopic images of ICC stained with MAP2 and GFAP antibodies show marked reduction in dendritic development (arrows) in cells treated with AraC from DIV3. (B,C) Quantification of MFI show decrease in MAP2 fluorescence (B) in KCl treated cells with late AraC (from DIV9) treatment (C). Note the increase in GFAP expression level with early to late AraC treatment regimens (B). Scale bar 100 μm. [file Image_2.jpeg]

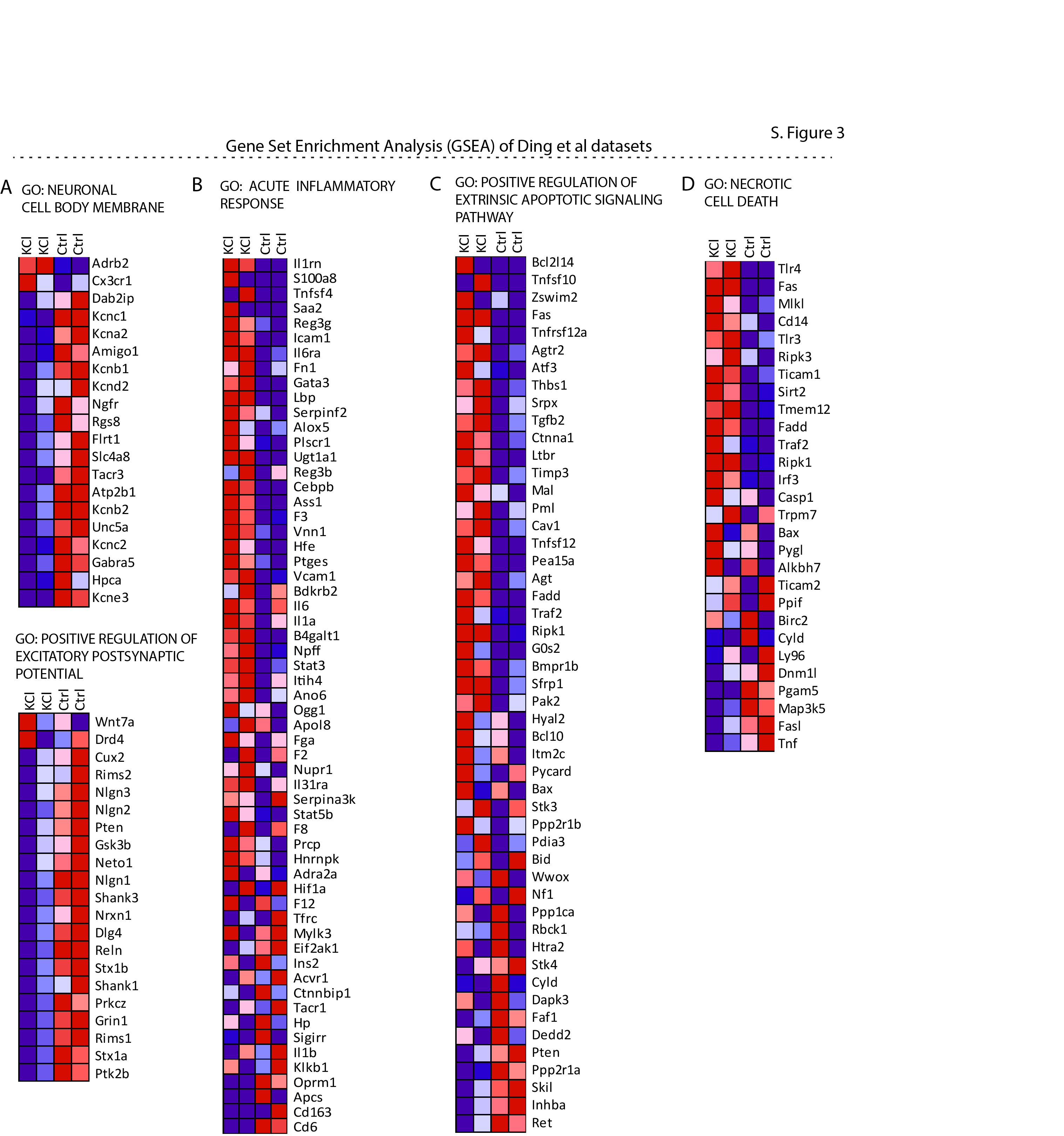

Supplement: Supplementary Figure 3 — (A) Heatmaps from Gene Set Enrichment Analysis (GSEA) of Ding et al. datasets show de-enrichment for several genes encoding neuronal cell body membrane and positive regulators of excitatory postsynaptic potential in KCL treated compared to control neurons. (B) Enrichment of inflammatory response genes and (C,D) postiive regulators of apoptosis and necrosis in KCL treated compared to control neurons. [file Image_3.jpeg]

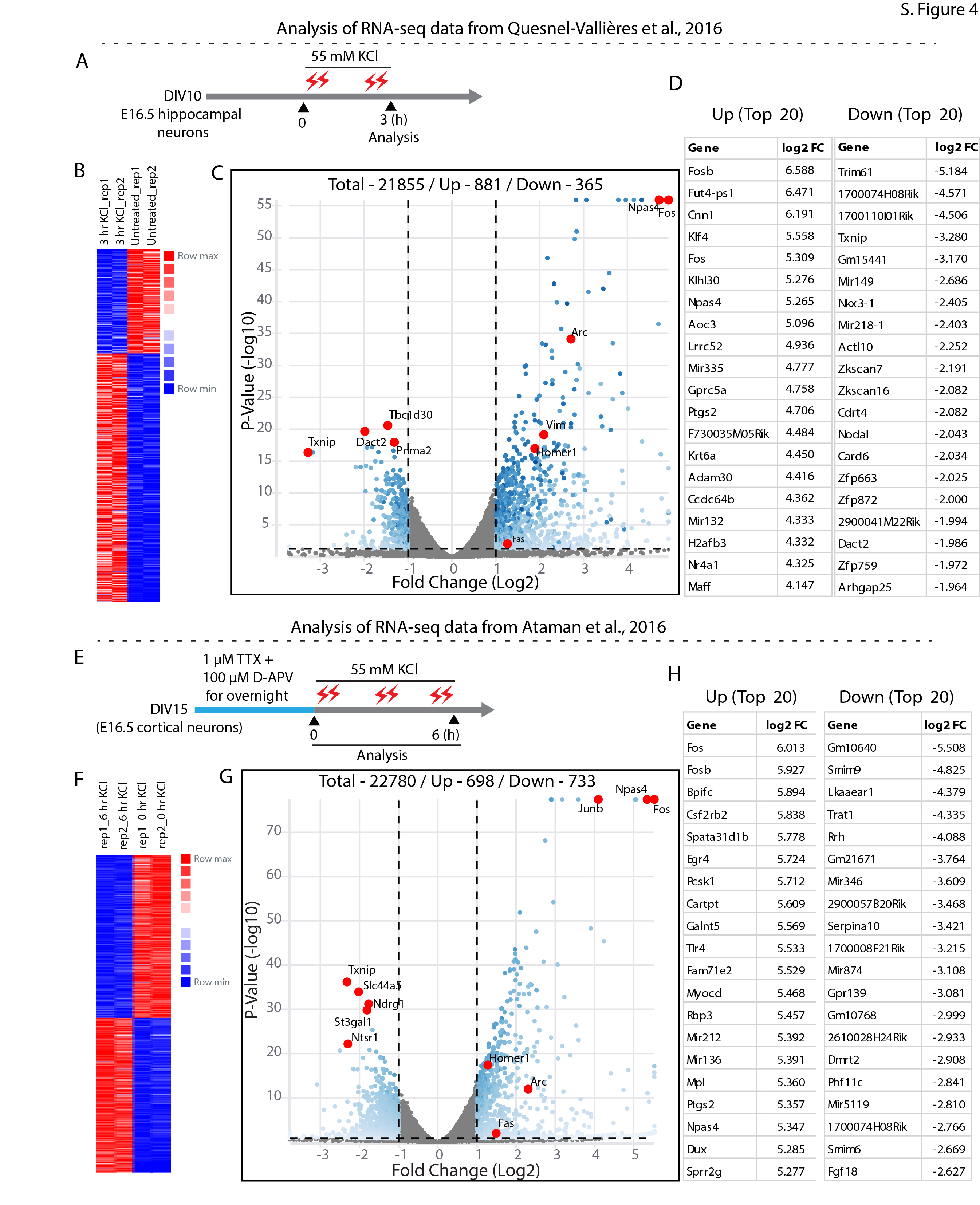

Supplement: Supplementary Figure 4 — RNA-seq analysis of Quesnel-Vallières et al., and Ataman et al. datasets. (A) Experimental paradigm used in Quesnel-Vallières et al. study, where DIV10 hippocampal neurons isolated from E16.5 mouse embryos chronically exposed to 55 mM KCl and samples collected at 3 h for RNA-seq analysis. (B) Heatmap show differential gene expression between duplicate KCl and untreated control samples (C) Valcano plot showing Log2 fold change of up and down regulated genes. Note significant upregulation of IEGs, Npas4, cFos, and Arc. No de-enrichment for neuronal markers found. There is an onset of upregulation of Fas, a pro-apoptotic gene. (D) List of top 20 up and down regulated genes. (E) Experimental paradigm used in Ataman et al. study, where DIV15 cortical neurons isolated from E16.5 mouse embryos were silenced using 1 μM TTX + 100 μM D-APV for overnight before chronically exposing to 55 mM KCl or NaCl. Samples collected at 0 and 6 h for RNA-seq analysis. (F) Heatmap show differential gene expression between duplicate KCl and 0 h control samples. (G) Valcano plot showing Log2 fold change of up and down regulated genes. Note significant upregulation of IEGs, Npas4, Fos, Junb, and Arc. No de-enrichment for neuronal markers found. There is an onset of upregulation of Fas, a pro-apoptotic gene. (H) List of top 20 up and down regulated genes. [file Image_4.jpeg]

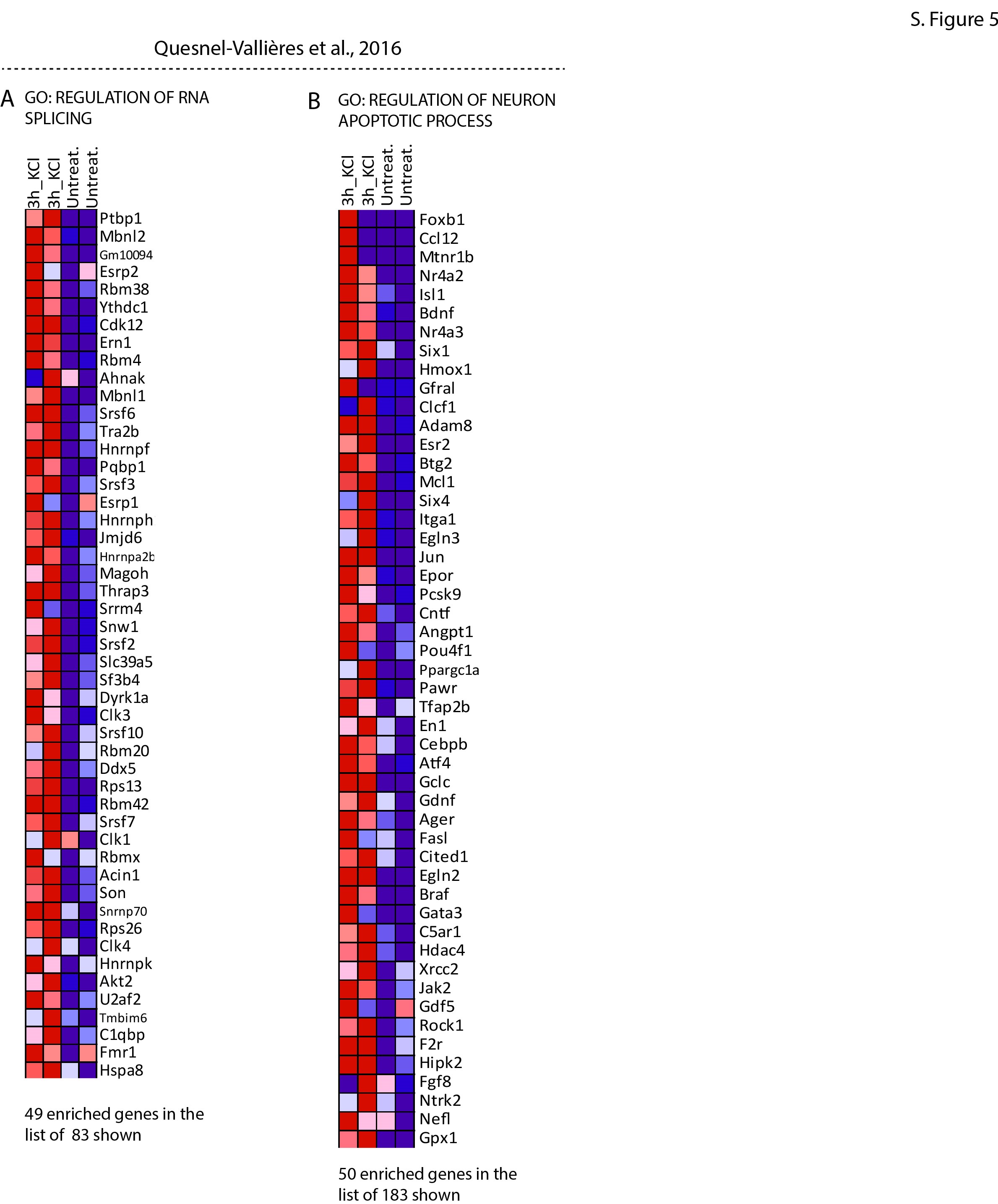

Supplement: Supplementary Figure 5 — GSEA analysis of Quesnel-Vallières et al., datasets. (A) Heatmap from GSEA analysis show enrichment of 49 genes (of 83) in the GO category: regulation of RNA splicing in 3 h KCl treated cells compared to untreated cells. (B) GSEA heatmap shows 50 genes (of 183 in the GO category) that are in the regulation of neuron apoptotic process category. [file Image_5.jpg]

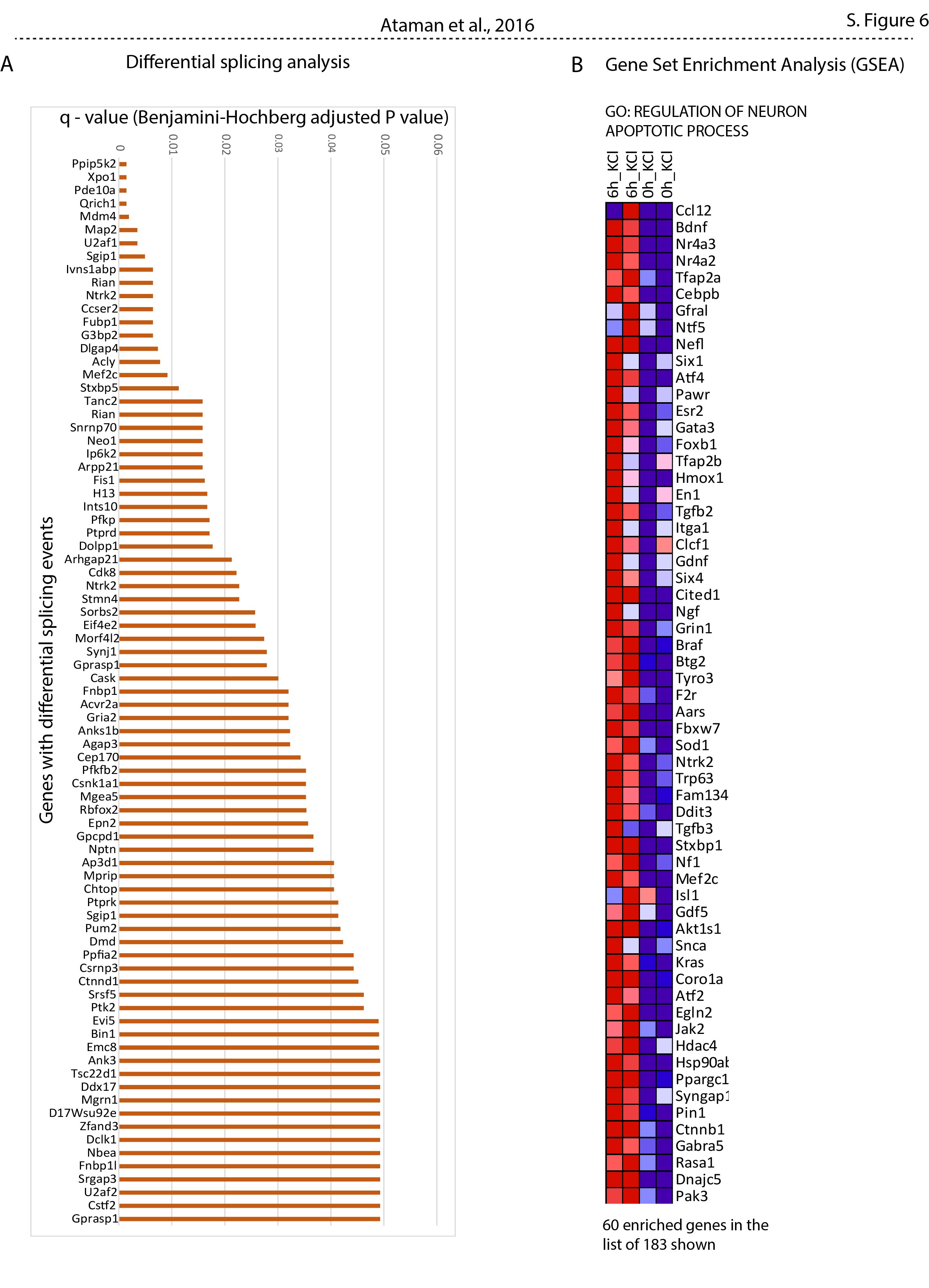

Supplement: Supplementary Figure 6 — Differential splicing and GSEA analysis from Ataman et al., datasets. (A) Bar graph shows genes with differential splicing events analyzed from the datasets. Note that Nrxns does not have any differential splicing events. (B) Heatmap from GSEA analysis show enrichment of 60 genes (of 183) in the GO category: regulation of neuron apoptotic process in 6 h KCl treated cells compared to 0 h treated cells. [file Image_6.jpg]
